# A Carbon‐Induced Surface Reconstruction on an Operating Fischer–Tropsch Catalyst

**DOI:** 10.1002/anie.5104870

**Published:** 2026-07-16

**Authors:** Sebastian Kläger, Sung Sakong, Axel Groß, Joost Wintterlin

**Affiliations:** ^1^ Department of Chemistry Ludwig‐Maximilians‐Universität München Munich Germany; ^2^ Institute of Theoretical Chemistry University of Ulm Ulm Germany; ^3^ Center for NanoScience Munich Germany

**Keywords:** Co(0001), density functional calculations, Fischer–Tropsch synthesis, operando, scanning probe microscopy

## Abstract

Heterogeneous catalysts are often assumed to undergo substantial chemical and morphological changes under reaction conditions. However, obtaining information on the state of a catalyst surface at the usually applied high pressures is difficult. For the Co catalyst used in the industrial Fischer–Tropsch synthesis, a widely held view is that the surface experiences a carbon‐induced roughening that is essential for its activity. We have tested this idea by performing the reaction on a Co(0001) surface with a sacrificial carbide layer. In situ scanning tunneling microscopy experiments at ∼1 bar synthesis gas and 473 K show that, at the increased C coverage, the surface does not undergo the predicted roughening. Instead, a unique carbon‐induced reconstruction forms, consisting of triangular partial stacking faults in which C atoms occupy the fault lines. Density functional theory calculations reveal that the reconstruction is more stable than the roughened state. For the detectable range of C_1_ to C_4_ reaction products, gas chromatography shows no effect of the reconstruction, consistent with the view that the activity is primarily determined by the density of atomic steps, which remains unchanged. These findings can serve as a new basis for describing the Co particles of the industrial catalyst.

## Graphical abstract

It is widely believed that a cobalt Fischer–Tropsch catalyst initially undergoes an activity‐enhancing roughening, driven by the high chemical potential of carbon. In this *operando* STM and DFT study, we simulate such conditions. We show that, instead, a triangular stacking‐fault reconstruction may form, leaving the catalytic activity unchanged.

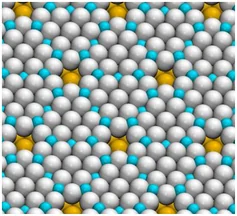

## 1 Introduction

Surfaces of supported catalysts under industrial reaction conditions can differ significantly from the single‐crystal models used in ultra‐high vacuum (UHV) experiments. At the high pressures applied in industrial processes, adsorbate coverages are much higher than in UHV experiments at the same temperature. Surface morphologies can be affected by the high coverages of adsorbates, and even chemical surface compounds can be formed. A known example is the reaction of CO with O_2_ on several Ru, Pd, and Pt single‐crystal surfaces, which, at an increased pressure, takes place on thin oxide films rather than on the metallic surfaces, which significantly enhances the catalytic activity [1, 2, 3].

The Co‐catalyzed Fischer–Tropsch synthesis is an industrial process for which there is evidence that the surface conditions on the actual catalyst are, in fact, not captured by existing model studies. In the industrial process, synthesis gas (syngas) at a ∼2:1 ratio of H_2_ and CO, at *p* = 20–40 bar, and *T*∼500 K, reacts over an oxide‐supported metallic Co catalyst to give linear paraffins and olefins: [4]

$$\left(2n+1\right)\;H_{2}+n\mathrm{CO}\;\to \;C_{n}H_{2n+2}+nH_{2}O,$$

$$2nH_{2}+n\mathrm{CO}\;\to \;C_{n}H_{2n\;}+nH_{2}O$$

Currently, the reaction is used to produce synthetic fuels on a large scale, and in the future, it may play an essential role in defossilizing hard‐to‐electrify mobility sectors. In several model studies on various Co single‐crystal surfaces, it has been achieved to perform experiments at gas pressures up to 1 bar (in one case even at a syngas partial pressure of 2.4 bar) and at temperatures around 500 K, conditions that come close to the industrial process [5, 6, 7, 8, 9, 10, 11, 12]. However, there is evidence that even under these elaborate conditions, a critical difference to the industrial process remains, and that adsorbed carbon atoms are responsible for this discrepancy.

Among the various CO activation mechanisms discussed in the literature (none of which may be the sole mechanism involved), the formation of C atoms through the dissociation of CO at defect sites is widely accepted as a key step: [13, 14]

$$\mathrm{CO}\;\to \;C_{\mathrm{ad}}+O_{\mathrm{ad}.}$$

At the pressures of the industrial reaction, this process must lead to a higher steady‐state coverage of C_ad_ than at ∼1 bar. This assumption is confirmed by the production of long hydrocarbon molecules in the industrial process [4], whereas in the model studies only C_1_ to C_4_ hydrocarbons are obtained [5, 6, 7, 10, 11, 12]. At a higher C_ad_ coverage, reactions of C atoms with hydrogen give a higher coverage of CH_x_ intermediates, and, consequently, faster polymerization. A high carbon coverage is also indicated by the carbon compounds that build up on Fischer–Tropsch catalysts with increasing process time. Bulk Co carbide (Co_2_C), polymeric carbon, and graphitic carbon have been detected [15, 16, 17, 18, 19, 20, 21, 22]. While their formation indicates a high chemical potential of carbon, none of these species has been observed in the model studies under in situ conditions, consistent with a low C coverage [10, 11, 12, 23].

In the community, there is a widespread idea that at a high coverage of C atoms, the structure of the Co surface changes, and that this restructured surface represents the active state of the catalyst. The idea originated from an early scanning tunneling microscopy (STM) study that showed, albeit by an ex situ experiment, that an initially flat Co(0001) sample had become rough after exposure to 4 bar of syngas at 523 K [24]. Observations that fresh, but already reduced, supported Co catalysts showed an increasing activity over a period of several days were accordingly attributed to roughening processes at the surface of the Co particles that led to a higher concentration of active sites [25]. Theory work explained these observations by the formation of small, monolayer‐high islands of Co atoms that are stabilized by adsorbed C atoms and CO molecules at the rims [26]. Another explanation has been the formation of a 2D carbide phase during the reaction. It has a lower Co density than the metal, so that during formation of the carbide Co atoms are ejected from the top layer and condense to give small islands [27]. Both explanations are based on an increased C coverage at the high pressure of the industrial process. In situ (though not *operando*) STM data have been claimed to show such islands [26]. As it is generally expected that atomic steps are the active sites in the Co‐based Fischer–Tropsch synthesis, such a carbon‐induced roughening could explain the activity of the catalyst.

Our previous in situ STM studies, which were combined with activity measurements (representing *operando* conditions), provided direct proof that atomic steps are indeed the active sites [10, 11, 12]. However, the claimed morphology changes have not been observed, despite extensive series of experiments at syngas pressures up to ∼1 bar and temperatures of 473–513 K. The samples were catalytically active and showed central characteristics of Fischer–Tropsch selectivity and kinetics, but the surface morphology was indistinguishable from that in vacuum. Furthermore, the measured activities per surface atom could be extrapolated to those of unpromoted nanoparticle‐based catalysts (>8 nm particle size), leaving little room for additional morphological effects in the remaining pressure gap. Nevertheless, it has been argued that the chemical potential of adsorbed C atoms, the major driving force for the predicted restructuring, has been too low to induce roughening at the applied pressures [14]. From the available data, this cannot be ruled out.

In sum, it is not clear whether the industrial Fischer–Tropsch catalyst only has a higher carbon coverage than the surfaces in the model experiments, or whether there is a qualitative, possibly structural, difference that is essential for the activity. Expanding *operando* experiments in model studies to significantly higher pressures than 1 bar is beyond current experimental possibilities [28, 29, 30].

Here, we present a study in which we have approached this problem indirectly. We prepared carbide layers on a Co(0001) crystal and performed the Fischer–Tropsch synthesis at ∼1 bar syngas with the carbide‐covered sample. Most of the carbide quickly reacts with hydrogen at the start of the reaction, but a relatively high fraction of the C atoms remains on the surface, modeling an increased chemical potential of carbon. The experiments thus simulate, at least transiently, the conditions on the surface of a Co catalyst at a higher syngas pressure. Using in situ STM, the evolution of the surface structure was monitored, turnover frequencies (TOFs) were measured by gas chromatography (GC), and the energetics of the surface were evaluated by density functional theory (DFT). We find that the surface structure indeed changes due to the effect of C atoms. However, it does not form the predicted roughened state but a unique, more stable reconstruction. The data may resolve the contradictions about the role of carbon on the active surface.

## 2 Results and Discussion

### 2.1 Preparation of the Surface Carbide

**FIGURE 1:**
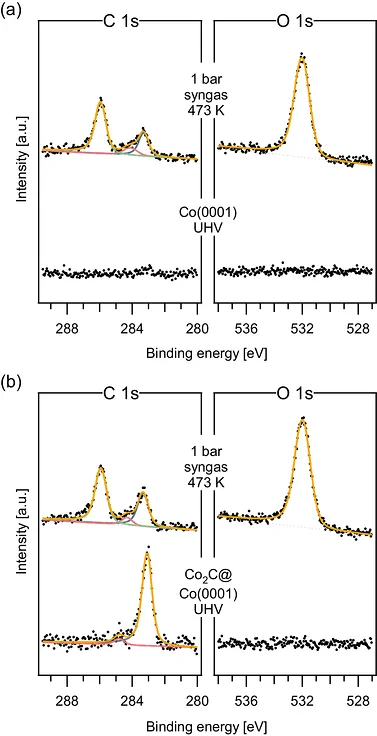
XP spectra of the Co(0001) surface. (a) C 1s and O 1s regions after preparation in UHV (bottom) and after reaction in 950 mbar syngas, 473 K, for 4 h (top). (b) Spectra after preparation of the carbide layer (bottom) and after reaction of the carbide‐covered sample in 950 mbar syngas, 473 K, for 4 h (top). The shift of the C 1s peak at 283.3 eV in the spectra after the reaction [(a) and (b), top], which is attributed to CH_x_, with respect to the peak at 283.1 eV of the carbide [(b), bottom], is larger than the experimental uncertainty.

After cleaning the Co(0001) crystal in the UHV chamber of the apparatus, carbide layers were prepared by dosing 10–20 Langmuirs (L, 1 L = 1.33 × 10^−6^ mbar × s) of ethylene at 623 K, and then holding this temperature for an additional 5 min. Carbon atoms from the ethylene decomposition remain on the surface, while molecular hydrogen desorbs [31, 32]. On the clean sample, X‐ray photoelectron spectroscopy (XPS) shows a carbon signal at the detection limit and no oxygen signal (Figure 1a, bottom). After dosing ethylene, XP spectra show a narrow C 1s peak at 283.1 eV, which is assigned to carbide (Figure 1b, bottom) [33]. Carbon coverages were between 0.40 and 0.46 monolayers [ML, defined as C atoms per surface Co atom], independently of dosage within the tested range, indicating that the surface was saturated with carbon.

**FIGURE 2:**
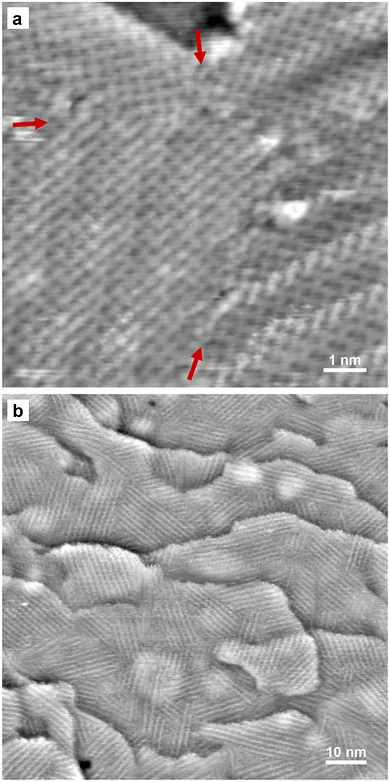
STM images of the carbide‐covered Co(0001) surface, taken at room temperature in UHV. (a) Small‐scale image; arrows indicate the directions of domain boundaries, tunneling voltage (*V*_t_) = −0.1 V, tunneling current (*I*_t_) = 7 nA. (b) Large‐scale image, *V*_t_ = −0.7 V, *I*_t_ = 0.7 nA.

STM images of the carbon‐saturated surface in UHV at room temperature show a nearly square atomic pattern (Figure 2a). It forms rotational and mirror domains, three of which can be seen in Figure 2a. STM images of larger areas (Figure 2b) show a pattern of stripes that run along six different directions, corresponding to six domains.

We interpret this pattern as a single layer of cobalt carbide, Co_2_C. Similar patterns have been reported before [31, 34], but a structural analysis is unavailable so far. However, there is a so‐called clock reconstruction model for a carbide phase formed on the Ni(111) surface [35, 36]. It features a single, probably incommensurate, Ni_2_C layer in which C atoms are coordinated by four Ni atoms in square–planar arrangements. The C coverage in the ideal structure is 0.44 ML. The square atomic lattice observed here on the Co(0001) surface, the six rotational and mirror domains possible from symmetry, and the C coverage (0.40–0.46 ML) match the Ni_2_C layer on Ni(111) very well.

The surface morphology—moderately wide atomic terraces mainly separated by monoatomic steps (Figure 2b)—is the same as on the bare Co(0001) sample that has been investigated in previous studies [8, 10, 37]. When the carbide layer is prepared, considerable rearrangements must occur because of the different Co densities in the carbide and metal layer. However, at the preparation temperature of 623 K, these processes are apparently fast enough to heal any morphology changes. The low, round hillocks are lattice deformations caused by Ne ions implanted into the bulk during sputter cleaning; these defects are also observed on the bare surface [8].

### 2.2 Evolution of the Carbide‐Covered Surface Under Fischer–Tropsch Conditions

**FIGURE 3:**
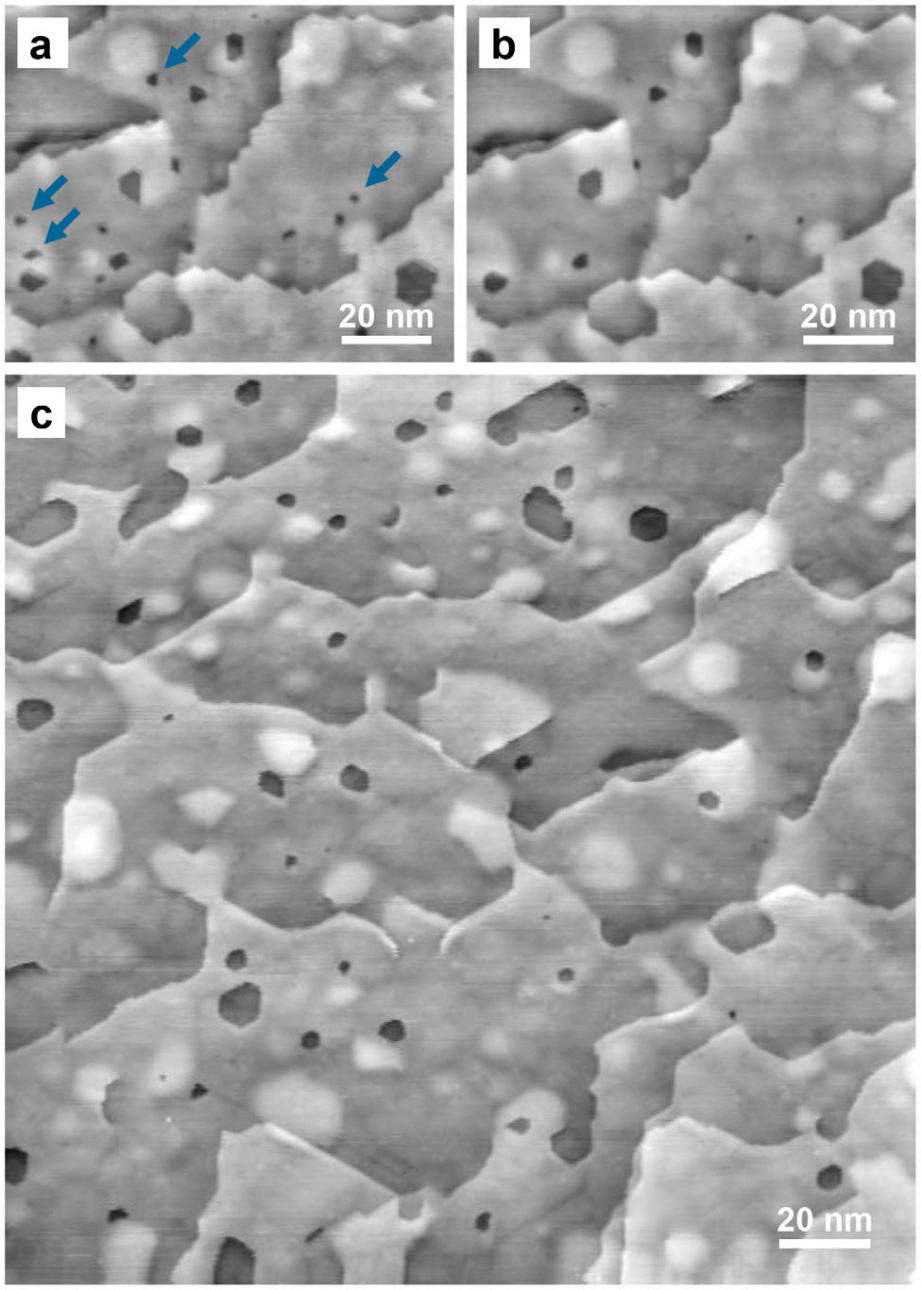
STM images of the initially carbide‐covered Co(0001) surface recorded in 950 mbar syngas at 473 K, after the carbide has reacted with hydrogen, *V*_t_ = −0.2 V, *I*_t_ = 2.3 nA. (a),(b) Images recorded 1:00 h and 1:45 h after starting the reaction; blue arrows in (a) mark holes that have disappeared in (b). (c) Large‐scale image of the same area, recorded 3 h after starting the reaction.

After preparation of a carbide layer, the STM/reactor cell of the apparatus was filled with 950 mbar syngas, and the sample temperature was increased to 473 K. Fischer–Tropsch products were then reproducibly detected by the attached GC. STM images taken in situ show that the stripe structure has completely disappeared (Figure 3). Under reaction conditions, the carbide structure is obviously unstable and disintegrates by reaction with hydrogen. The resulting surface displays almost flat monoatomic terraces. This morphology is the same as observed in previous studies on Co(0001) and Co$\left(10\bar{1}15\right)$ surfaces that were performed under such conditions but started with the metallic surfaces [10, 11, 12]. The only difference is the many small and mostly monolayer‐deep holes that only occur in the experiments with the carbide‐covered surface. Figure 3a,b, taken 1:00 and 1:45 h after starting the reaction, shows that the holes shrink and start to disappear (blue arrows) on the time scale of the experiment. In Figure 3c, the holes cover 3%–4% of the surface area. Healing of surface defects by mass transport of Co atoms under such conditions has been demonstrated before [10].

The topographic holes are initially formed because the density of Co atoms in the starting Co_2_C phase is lower than in a metal layer. Assuming the same structure model as for the Ni_2_C clock reconstruction, the estimated coverage of Co atoms in the carbide layer is 2 × 0.44 ML = 0.88 ML, that is 12% lower than in a metal layer. Hence, when the carbide layer disintegrates, the surface layer contains too few Co atoms to form a complete monolayer of the metal. A fraction of the lacking material appears in the form of holes, and another fraction is explained by a reduced Co atom density in the newly formed surface phase (see below).

The disintegration of the carbide is fast. We attempted to capture the process by working at a relatively low temperature (473 K), the lower limit at which catalytic activity could still be measured. However, the stripe structure had already disappeared in the first stable STM snapshots recorded ≥20 min after setting the sample temperature for the reaction. Exposure of the carbide to syngas at room temperature causes a disordering of the clock reconstruction even at a pressure of ∼1 × 10^−8^ mbar and leads to a fully amorphous structure at 950 mbar (Figure S1). After the carbide has disintegrated, there are also gradual changes over time in the orientations of steps (Figure S2).

**FIGURE 4:**
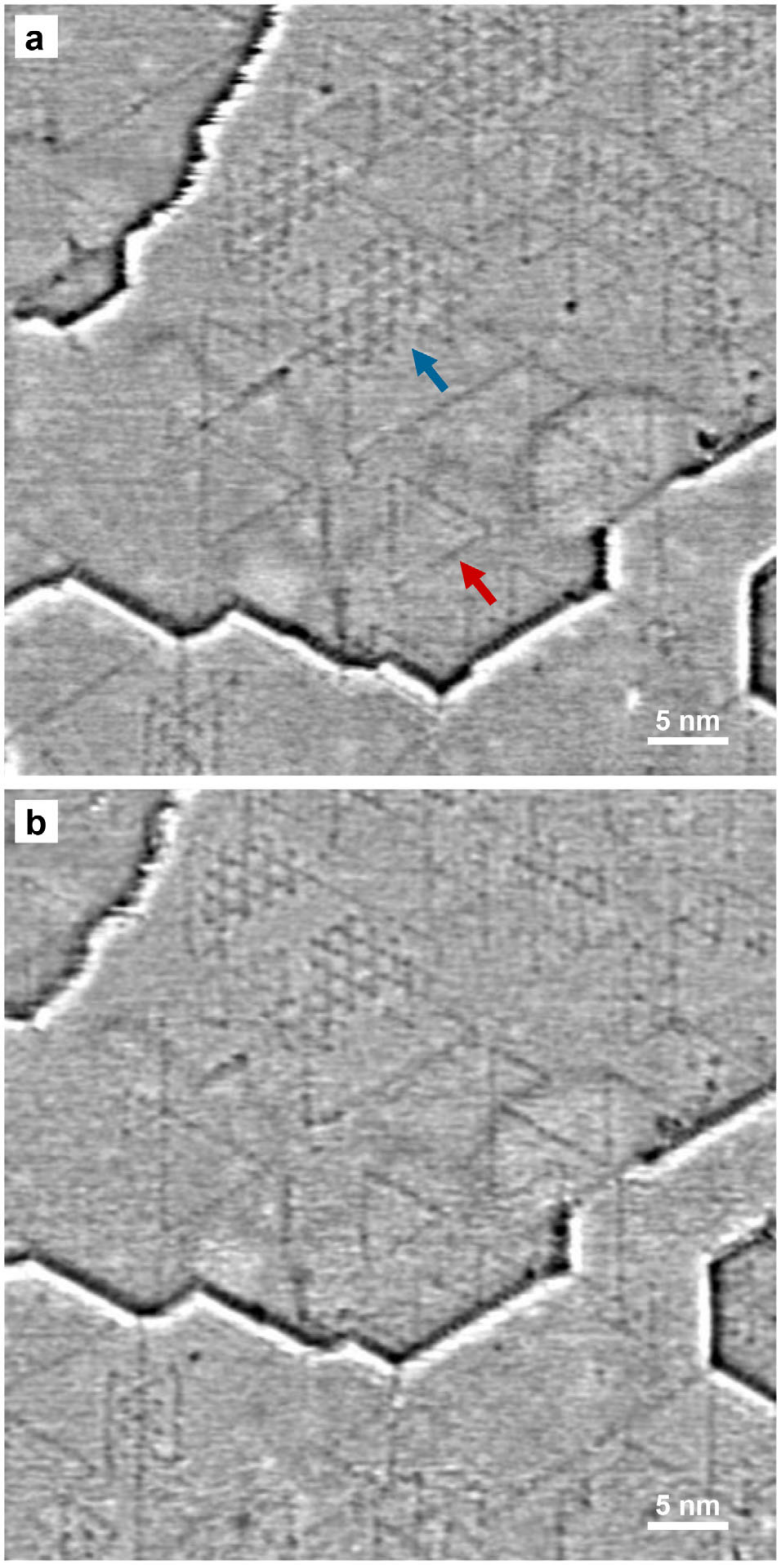
STM images of the initially carbide‐covered Co(0001) surface with enhanced contrast under reaction conditions (950 mbar syngas, 473 K), *V*_t_ = −0.2 V, *I*_t_ = 2.3 nA. (a) Image recorded 1 h after starting the reaction. (b) Image recorded on the same area 9 min after acquisition of the image in (a). The red arrow in (a) indicates a triangle that has become smaller in (b). The smallest triangles in the clusters in the upper parts of the images have side lengths of six lattice constants; one is marked by a blue arrow.

The instability of the carbide layer is consistent with reports that this surface phase does not form under the applied reaction conditions in experiments starting with a metallic Co surface [10, 11, 12, 23]. However, the present study differs from this previous result in one essential aspect. When the image contrast on the terraces formed by disintegration of the carbide layer is enhanced, a characteristic pattern of triangular features appears (Figure 4). Equilateral triangles of various sizes are visible, the smallest with side lengths of six Co(0001) lattice constants (blue arrow). The edges are oriented along the close‐packed directions of the metal. High coverages of these patterns were reproducibly observed in multiple experiments with carbon‐enriched surfaces. Previous experiments with the metallic surface have occasionally shown such features, which, however, only covered small parts of the surface [10], and after more extensive surface preparation, they were completely absent (Figure S3) [12]. In contrast to the shrinking topographic holes, the triangles are relatively stable over the time periods of the experiments; only minor changes were observed (the red arrow in Figure 4a shows a triangle feature that has shrunk in Figure 4b). Hence, when the reaction is started with the carbide‐covered surface, the carbon coverage quickly drops by reaction with hydrogen, but, as the triangular features are carbon‐induced (see below), it remains at a higher steady‐state level than in the experiments with the metallic surface.

Post‐reaction XP spectra were acquired ex situ, after several hours of reaction, but notably within the same apparatus to exclude air contact (Supporting Information, Experimental Section). Nevertheless, the chemical state of the surface certainly changed when, after finishing a reaction experiment, the sample cooled in syngas and was transferred to the UHV chamber after pumping down the STM cell. Probably C atoms became hydrogenated, and hydrogen and a fraction of CO desorbed. However, the spectra still contain valuable information, particularly when related to recently published in situ XPS data [23]. The spectra from the metallic and the carbide‐covered surfaces (Figure 1a,b, top) show C 1s peaks at 283.3 eV attributed to partially hydrogenated atomic carbon (CH_x_). The peaks at 284.3 eV can be attributed to polymeric carbon (0.04 ML), the peaks at 285.9 eV to adsorbed CO (0.24 and 0.27 ML on the initially metallic and carbide‐covered surfaces, respectively), and the O 1s peaks at 532.0 eV also to adsorbed CO (0.24 and 0.27 ML). All assignments align with recent in situ XPS results [23]. The presence of various C species at sub‐monolayer coverages reflects the situation under reaction conditions, even though the coverages are certainly altered. That the C 1s and O 1s peaks in each case give identical CO coverages rules out significant quantities of OH or other oxygen species, also in agreement with the ambient‐pressure XPS study [23]. Moreover, other oxygen species, such as alcohols, appear at significantly lower O 1s binding energies [38]. The only significant difference between the two types of experiments presented here is the higher coverage of CH_x_ on the initially carbide‐covered surface (0.16 ML) than on the initially metallic surface (0.10 ML). Since measurement and transfer conditions are identical, the relative difference confirms a higher steady‐state carbon coverage in the carbide experiment. No difference was found in survey spectra and in the Co 2p region (Figure S4).

The fact that the triangular structures were only observed in the experiments on the carbide‐covered surface and were absent on the bare surface shows that they are induced by a fraction of the C atoms released during the disintegration of the carbide layer. This is confirmed by the higher carbon coverage detected by XPS after the carbide experiments.

### 2.3 DFT Analysis of the Triangular Features

To derive a structure model of the triangular features, the energetics of adsorbed C atoms on the flat surface, at the edges of triangular Co islands (the basis of the mentioned roughening idea) [26], and on a reconstructed surface were evaluated by DFT. A reconstruction model for the triangles had been proposed in a previous STM study in which C atoms were created by dissociation of CO on a Co(0001) surface (without H_2_ exposure) [34]. The suggested reconstruction model consisted of triangular pieces of lattice planes forming fcc stacking faults; C atoms would occupy the fault lines to the surrounding hcp‐stacked surface.

**FIGURE 5:**
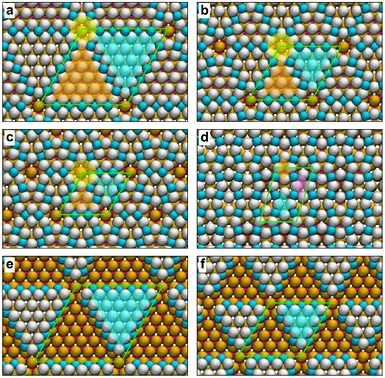
Optimized C‐induced structures on the Co(0001) surface obtained by DFT. Cyan balls represent C atoms, and white, yellow, and pink balls are Co atoms in the first, second, and third layers, respectively; green lines mark surface unit cells. Transparent shades in orange, blue, and pink mark Co atoms on fcc, hcp, and top sites, respectively; yellow shades mark vacancy sites. (a), (b), and (c) C‐induced triangular stacking‐fault reconstructions with (7 × 7), (5 × 5), and (4 × 4) unit cells, respectively. (d) Precursor configuration of the clock reconstruction. (e) and (f) Triangular Co islands with C atoms at the step edges with (7 × 7) and (5 × 5) unit cells, respectively.

**FIGURE 6:**
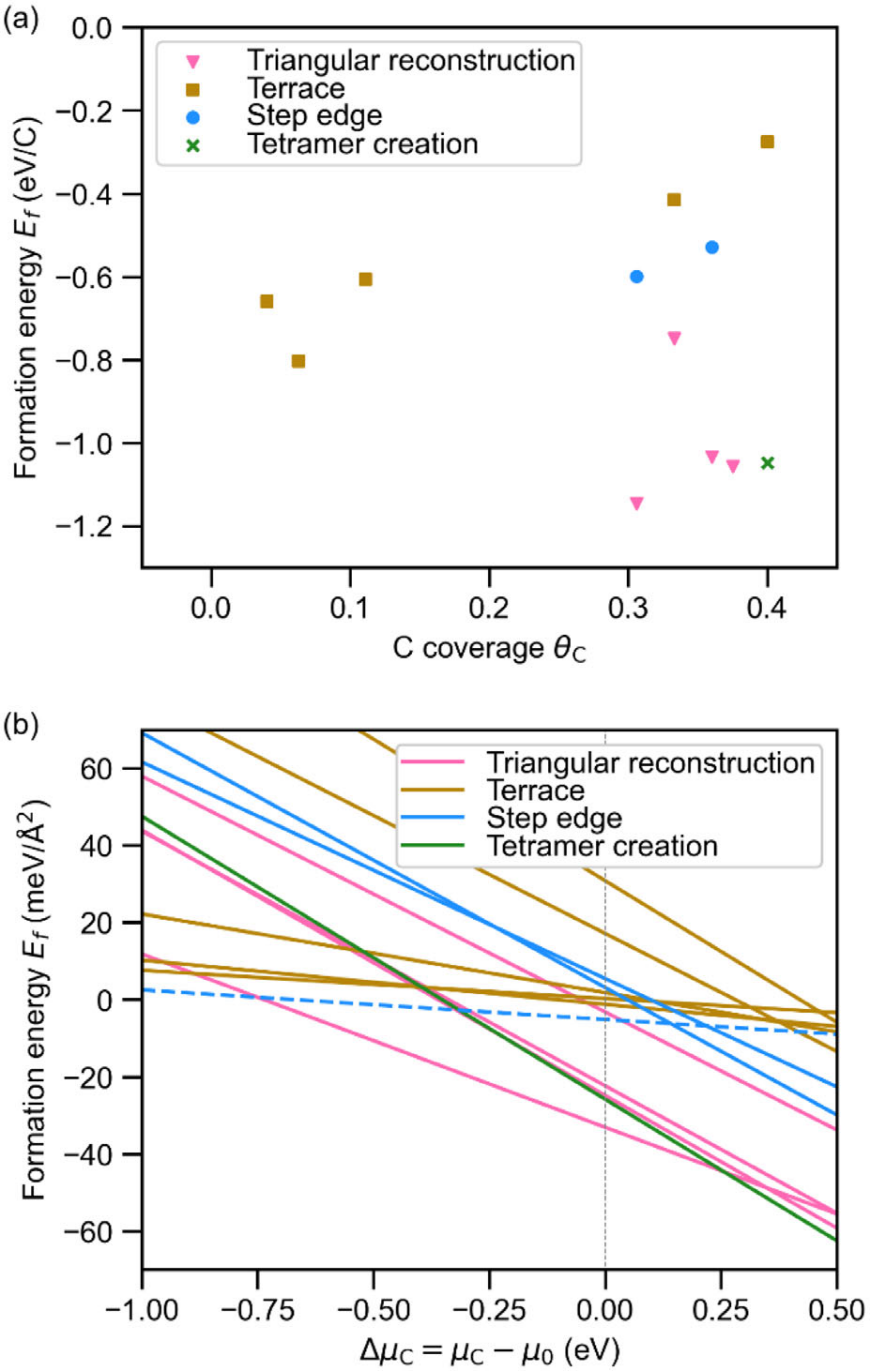
Formation energies obtained by DFT. (a) Formation energies of surface configurations with C atoms and varying numbers of Co atoms in the top layer. (b) Formation energies of surface configurations per unit area (*E*_f_ =  Δ*E*/*A*), indicating relative stabilities, as a function of the carbon chemical potential Δµ_C_. Δµ_C_ is relative to µ_0_, the chemical potential under typical Fischer–Tropsch synthesis conditions, 20 bar of syngas, 500 K, and 60% conversion.

We computed the formation energies *E*_f_ of the respective structures. Reconstructions with various unit cells were investigated. Figure 5a–c show (7 × 7), (5 × 5), and (4 × 4) unit cells of optimized models, with C atoms adsorbed at the boundaries between hcp‐ and fcc‐stacked areas. In the (7 × 7) and (5 × 5) structures, the C atoms alternatingly occupy sites above and below the top Co layer. Depending on positions, C atoms are coordinated by four or six Co atoms. C─Co bond lengths are around 2 Å. The C─Co bonds cause significant lateral strain, which explains why the Co atoms shift from hcp to fcc sites to optimize the C─Co bond lengths. Formation of the C─Co bonds in the reconstruction overcompensates for the energy cost to move Co atoms from hcp to fcc sites (25 meV/Co atom in the absence of carbon) and the energy cost to remove Co atoms from the close‐packed top layer (the reconstructed layer has fewer Co atoms). The triangle reconstructions (Figure 6a, pink triangles) clearly show a higher stability than adsorbed C atoms on the hcp sites of the flat surface (Figure 6a, brown squares). A peculiar feature of the reconstruction is the stable Co vacancy at the corner of the unit cell. We attempted to fill it with a Co atom, but it would occupy a top site position, which is unfavorable.

Figure 5e,f shows alternative models with triangular Co islands in a regular hcp stacking with respect to the underlying surface. We confirm the result from a previous study that C atoms adsorb strongly at the step edges at the rims of the islands [26]. As shown in Figure 6a (blue spheres) for (7 × 7) and (5 × 5) unit cells, the islands are, in fact, more stable than configurations with C atoms on the flat surface. However, the stabilization is weaker than for the stacking‐fault reconstruction because the island formation energy compensates to a considerable extent for the gain from adsorption at the favorable step sites. Adsorbed CO molecules stabilize the islands and the stacking‐fault reconstruction to about the same degree, so that the difference remains unchanged (Figure S5). Calculations of the thermodynamics in relation to the carbon chemical potential (Figure 6b) reveal that the stacking‐fault reconstruction remains preferred for a wide range of process parameters, including typical industrial conditions at µ_0_ (20 bar, 500 K, 60% conversion, same parameters as in ref [26].). The most stable structure under these conditions is the triangular stacking‐fault reconstruction (pink line) with a (7 × 7) unit cell. The carbon‐induced surface roughening (full blue lines) is less favorable under the conditions considered. The dashed blue line represents carbon adsorption at preexisting steps, which dominates below −0.75 eV. At carbon chemical potentials above 0.26 eV, tetramer creation is favored (the structure shown in Figure 5d, see description below).

In the reconstructed layer, the average density of Co atoms $\Theta_{\mathrm{Co}}^{\mathrm{rec}}$ is lower than in a close‐packed layer. $\Theta_{\mathrm{Co}}^{\mathrm{rec}}$ could be determined from the experiments by two methods, providing a consistency test. From the areas of the topographic holes, formed as a result of the density difference between the carbide and the reconstructed layers, $\Theta_{\mathrm{Co}}^{\mathrm{rec}}$ was determined by $\Theta_{\mathrm{Co}}^{\mathrm{rec}}=\Theta_{\mathrm{Co}}^{\mathrm{carb}}+\Theta_{\mathrm{holes}}$, with $\Theta_{\mathrm{Co}}^{\mathrm{carb}}$ = 0.88 ML and Θ_holes_ = 0.03–0.04 ML (Figure 3a). The value of 0.91–0.92 ML is a lower limit because the holes were shrinking with time. From the overall lengths of the fault lines, *L*_fault_, determined by statistically analyzing three large STM images, $\Theta_{\mathrm{Co}}^{\mathrm{rec}}$ could be evaluated by $\Theta_{\mathrm{Co}}^{\mathrm{rec}}=1-\left(\sqrt{3}/6\right)\left(L_{\mathrm{fault}}d/A\right)$, where *A* is the image area and *d* the Co(0001) lattice constant. The value of 0.96–0.99 ML is an upper limit because the corner holes of the triangles are neglected in this estimate, which is based on straight fault lines. The agreement of the two estimates is quite reasonable. As a further consistency test, the coverage of C atoms was evaluated from *L*_fault_. The obtained range, Θ_C_ = 0.03–0.11 ML, covers the XP‐spectroscopically measured difference between the carburized and the bare samples of ∼0.06 ML.

In the reconstruction model, triangles shrink with increasing C coverage, with the highest possible coverage of Θ_C_ = 0.375 ML for a triangle structure having a (4 × 4) unit cell (Figure 5c). When the C coverage is increased further in the calculations, the structure changes. Figure 5d shows the resulting model with three Co tetramers per unit cell (Θ_C_ = 0.40 ML), which has been created by shifting some Co atoms to top sites. Although one third of the Co atoms occupy these less stable sites, the formation energy (Figure 6a, green cross) is close to that of the (4 × 4) reconstruction. Note the alternating clockwise/anticlockwise rotations of the four Co atoms that coordinate the C atoms, which resemble the clock reconstruction of the surface carbide [35, 36]. In contrast to the known clock reconstruction, the structure is in registry with the substrate and 10% larger. Given the clock reconstruction's characteristic mismatch with the underlying close‐packed Co lattice, a lateral contraction would lead to a further stabilization by 0.25 eV/C. The triangle reconstruction can thus be interpreted as an intermediate stage in the formation of the surface carbide.

### 2.4 Activity and Selectivity Measurements

**FIGURE 7:**
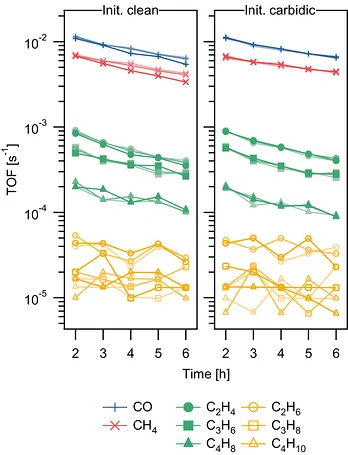
Turnover frequencies on the initially metallic surface (left) and on the initially carbide‐covered surface (right); 950 mbar syngas, 493 K, time period 6 h. Plotted are the formation rates of methane and of the C_2_─C_4_ paraffins and olefins, as well as the CO consumption rates (calculated from the hydrocarbon formation rates). The data points at 1 h were discarded because of slow temperature equilibration. The tabulated data plus the data at 473 K are given in Tables S1 and S2.

Effects of the reconstruction on the Fischer–Tropsch activity were studied in separate GC measurements, but in the same STM/reactor cell and under identical conditions. Like in the STM experiments, the reaction was carried out in 950 mbar syngas at 473 K, and, for higher product signals, also at 493 K. Chromatograms show signals for C_1_─C_4_ hydrocarbons (Figure S6); the signals for longer hydrocarbons were below the detection limit (the usual limitation of single‐crystal studies). TOFs expressed as CO consumption rates per Co atom were calculated from the increases in the produced hydrocarbons. Figure 7 shows data taken at 493 K. On the initially carbide‐covered surface, the average CO consumption rate is initially 1.12 × 10^−2^ s^−1^ and then drops to 0.65 × 10^−2^ s^−1^, and on the initially metallic surface, it drops from 1.13 × 10^−2^ to 0.61 × 10^−2^ s^−1^. Surprisingly, the TOFs from both surface states are almost identical within the (very low) scatter of the data. The TOFs of the individual hydrocarbons, and thus the selectivities within the experimentally accessible range of C_1_ to C_4_ products, are also almost identical. Why all activities gradually decrease over time has not been clarified so far, but has been reported before [11], and is also equally observed in both experiments. At 473 K, TOFs are lower, as expected, but still similar on the two surfaces (Tables S1 and S2).

Considering the usually observed, strongly reduced activity of Co bulk carbide [20, 39, 40], one may have expected a decreased activity of the C‐induced reconstruction, but this is not the case. This observation can plausibly be explained by the fact that the step density is unaffected by the reconstruction. Atomic steps are the dominant active sites for the dissociation of CO plus, possibly, for the O + H → OH reaction, the two reaction steps with the highest degrees of rate control [41, 42, 43]. The fact that the topographic holes have no measurable effect on the activity is explained by the relatively small increase in step sites (20% in the image of Figure 3c). The hardly changed step density can also explain the previous report that most published TOF values on unpromoted supported catalysts, even at high pressures (after proper parameter normalization), are in surprisingly good agreement with TOFs on a Co$\left(10\bar{1}15\right)$ crystal, which shows no roughening under reaction conditions and has a step density similar to that of Co nanoparticles of the industrial catalyst [11]. Whether the C atoms in the reconstruction contribute to the reaction, we cannot decide. The fact that the triangle features do not change with time might represent a steady‐state situation, but in this case, one would expect to see fluctuations of the triangle features. The red arrow in Figure 4 shows a rare example.

### 2.5 Implications for Real Fischer–Tropsch Systems

Given the model nature of this study, it is important to clarify which conclusions about the actual Fischer–Tropsch process can and cannot be drawn. We first distinguish between simple supported, unpromoted catalysts on non‐reducible supports, and more complex catalyst formulations. In the former case, there is a general consensus that metallic cobalt is the active phase [13]. Past model studies using single‐crystal Co samples have shown that the materials gap is smaller than generally assumed, with central characteristics of Fischer–Tropsch catalytic behavior being reproduced by a Co(0001) crystal [7, 10, 11, 12]. This includes compliance with Anderson–Schulz–Flory kinetics, including the typical deviations for the C_1_ and C_2_ hydrocarbons, the variations in the olefin‐to‐paraffin ratio with carbon chain length, and an apparent activation energy of ∼100 kJ mol^−1^. Furthermore, it has been shown that the activity of a stepped single‐crystal surface closely reproduces that of “simple” supported catalysts [11]. The primary surface processes on simple supported catalysts are therefore adequately accounted for by the model used here. Remaining differences may be explained by secondary effects like increased readsorption caused by liquid‐filled pores in supported catalysts [44]. The reconstruction found in the present study could therefore be transferable to a simple supported catalyst. The facets of Co nanoparticles consist of a considerable fraction of close‐packed terraces separated by monoatomic steps [45], so that these facets probably behave similarly to the Co(0001) surface investigated here.

The situation differs with more complex catalyst formulations, where additional active phases, such as cobalt oxides [46] or carbides [47, 48], develop, and the interfaces between various phases [49] and/or the support [46, 50] come into play. Strong metal–support interactions and promoters stabilize such conditions [13]. Clearly, under such circumstances, the C‐induced reconstruction might be obscured, and other morphological influences not accounted for by the present model can become dominant.

## 3 Conclusion

The surface of a Fischer–Tropsch catalyst under industrial conditions is characterized by a high chemical potential of adsorbed carbon. However, the pressures exceed the current limits of model studies. To simulate such conditions, the coverage of C atoms on a Co(0001) sample was artificially increased by the disintegration of a carbide layer. Under Fischer–Tropsch reaction conditions (1 bar syngas, 473 K), a characteristic carbon‐induced surface reconstruction was found. The reconstruction is formed by triangular fcc stacking faults of the hcp‐stacked metal surface, where the fault lines provide adsorption sites for the C atoms. DFT calculations show that, at a given C coverage, the reconstruction is more stable than Co monolayer islands with adsorbed C atoms at the step sites. The reconstructed state is stable over several hours of the reaction, and it shows the same Fischer–Tropsch activity as the unreconstructed, flat surface. These results allow us to reevaluate existing views about the morphological effects of industrial reaction conditions.

At syngas pressures of ≥20 bar, where the steady‐state coverage of C atoms must be considerably higher than in the model studies, the surface layer of supported catalyst nanoparticles may display a C‐induced surface reconstruction, especially if the support is non‐reducible and there is no promoter. The reconstruction can be considered an intermediate stage of surface carbide formation. Its presence does not significantly affect the density of atomic steps, and no effect on the overall catalytic activity was observed. This finding aligns with previous results from model studies that atomic steps determine the catalytic activity. Of course, additional phenomena, such as the effect of oxygen, can come into play at higher pressures, and promoter effects or strong metal–support interactions in real systems may override the reconstructing effect of carbon.

Our findings contradict a C‐induced formation of Co monolayer islands, a widespread concept to explain the catalytic activity. No such islands have been observed at the increased C coverage, and the calculations show that the reconstruction is more stable than islands (for the relatively small supercells considered here). The initial activity increase observed for supported catalysts must be explained differently, possibly by promoter effects or metal–support interactions [51, 52].

## Author Contributions

**Sebastian Kläger**: investigation, writing – original draft, methodology, validation, visualization, conceptualization, writing – review and editing. **Sung Sakong**: investigation, writing – original draft, methodology, validation, visualization, software, conceptualization, writing – review and editing. **Axel Groß**: funding acquisition, writing – review and editing, supervision. **Joost Wintterlin**: conceptualization, funding acquisition, writing – original draft, writing – review and editing, supervision, methodology, project administration.

## Conflicts of Interest

The authors declare no conflicts of interest.

## Supporting information




**Supporting File**: Please see the Supporting Information for the Experimental Section, Computational Methods, and Supporting Figures and Tables: STM images of the carbide‐covered Co(0001) surface under syngas at room temperature (Figure S1), STM images showing the changing orientations of step edges under reaction conditions (Figure S2), STM images of the originally bare Co(0001) sample under reaction conditions (Figure S3), XP spectra of the Co 2p region and survey spectra (Figure S4), models of reconstructed surfaces with adsorbed CO as obtained by DFT (Figure S5), gas chromatograms (Figure S6), and tabulated activity data (Tables S1 and S2). Additional references cited within the Supporting Information [53, 54, 55, 56, 57, 58, 59, 60, 61, 62, 63].

## Data Availability

The data that support the findings of this study are openly available in Zenodo at https://doi.org/10.5281/zenodo.20292451.

## References

[1] H. Over , Y. D. Kim , A. P. Seitsonen , et al., “Atomic‐Scale Structure and Catalytic Reactivity of the RuO_2_(110) Surface,” Science 287 (2000): 1474–1476, 10.1126/science.287.5457.1474. 10688793

[2] B. L. M. Hendriksen , S. C. Bobaru , and J. W. M. Frenken , “Oscillatory CO Oxidation on Pd(100) Studied With In Situ Scanning Tunneling Microscopy,” Surface Science 552 (2004): 229–242, 10.1016/j.susc.2004.01.025.

[3] B. L. M. Hendriksen , S. C. Bobaru , and J. W. M. Frenken , “Looking at Heterogeneous Catalysis at Atmospheric Pressure Using Tunnel Vision,” Topics in Catalysis 36 (2005): 43–54, 10.1007/s11244-005-7861-7.

[4] C. H. Bartholomew and R. J. Farrauto , Fundamentals of Industrial Catalytic Processes, 2nd ed. (Wiley‐Interscience, 2006), 398–486.

[5] J. J. C. Geerlings , M. C. Zonnevylle , and C. P. M. de Groot , “Studies of the Fischer‐Tropsch Reaction on Co(0001),” Surface Science 241 (1991): 302–314, 10.1016/0039-6028(91)90090-F.

[6] J. J. C. Geerlings , M. C. Zonnevylle , and C. P. M. de Groot , “Structure Sensitivity of the Fischer‐Tropsch Reaction on Cobalt Single Crystals,” Surface Science 241 (1991): 315–324, 10.1016/0039-6028(91)90091-6.

[7] G. A. Beitel , C. P. M. de Groot , H. Oosterbeek , and J. H. Wilson , “A Combined In‐Situ PM‐RAIRS and Kinetic Study of Single‐Crystal Cobalt Catalysts Under Synthesis Gas at Pressures up to 300 mbar,” Journal of Physical Chemistry B 101 (1997): 4035–4043, 10.1021/jp963337p.

[8] M. Ehrensperger and J. Wintterlin , “in situ High‐Pressure High‐Temperature Scanning Tunneling Microscopy of a Co(0001) Fischer–Tropsch Model Catalyst,” Journal of Catalysis 319 (2014): 274–282, 10.1016/j.jcat.2014.09.011.

[9] V. Navarro , M. A. van Spronsen , and J. W. M. Frenken , “in situ Observation of Self‐Assembled Hydrocarbon Fischer–Tropsch Products on a Cobalt Catalyst,” Nature Chemistry 8 (2016): 929–934, 10.1038/nchem.2613. 27657868

[10] B. Böller , K. M. Durner , and J. Wintterlin , “The Active Sites of a Working Fischer–Tropsch Catalyst Revealed by Operando Scanning Tunnelling Microscopy,” Nature Catalysis 2 (2019): 1027–1034, 10.1038/s41929-019-0360-1.

[11] K. M. Golder and J. Wintterlin , “In situ/*Operando* STM of the Fischer–Tropsch Synthesis on a Co(101̅15) Surface─a Study to Bridge the Materials Gap Between Single‐Crystal Models and Supported Catalysts,” ACS Catalysis 12 (2022): 7199–7209, 10.1021/acscatal.2c00703.

[12] S. Kläger , K. M. Golder , and J. Wintterlin , “Kinetics of the Fischer–Tropsch Synthesis on Cobalt Single Crystals Under Scanning Tunneling Microscopy Control,” ACS Catalysis 13 (2023): 14685–14698, 10.1021/acscatal.3c03050.

[13] I. C. ten Have and B. M. Weckhuysen , “The Active Phase in Cobalt‐Based Fischer‐Tropsch Synthesis,” Chemical Catalysis 1 (2021): 339–363, 10.1016/j.checat.2021.05.011.

[14] K. T. Rommens and M. Saeys , “Molecular Views on Fischer–Tropsch Synthesis,” Chemical Reviews 123 (2023): 5798–5858, 10.1021/acs.chemrev.2c00508. 36897768

[15] O. Ducreux , J. Lynch , B. Rebours , M. Roy , and P. Chaumette , “In situ Characterization of Cobalt Based Fischer‐Tropsch Catalysts: A New Approach to the Active Phase”, in: Studies in Surface Science and Catalysis, ed. A. Parmaliana D. Sanfilippo F. Frusteri A. Vaccari and F. Arena (Elsevier, 1998), 125–130, 10.1016/S0167-2991(98)80419-9.

[16] J. Xiong , Y. Ding , T. Wang , et al., “The Formation of Co_2_C Species in Activated Carbon Supported Cobalt‐Based Catalysts and Its Impact on Fischer–Tropsch Reaction,” Catalysis Letters 102 (2005): 265–269, 10.1007/s10562-005-5867-1.

[17] D. J. Moodley , J. van de Loosdrecht , A. M. Saib , M. J. Overett , A. K. Datye , and J. W. Niemantsverdriet , “Carbon Deposition as a Deactivation Mechanism of Cobalt‐Based Fischer–Tropsch Synthesis Catalysts Under Realistic Conditions,” Applied Catalysis A 354 (2009): 102–110, 10.1016/j.apcata.2008.11.015.

[18] H. Karaca , J. Hong , P. Fongarland , et al., “in situ XRD Investigation of the Evolution of Alumina‐supported Cobalt Catalysts Under Realistic Conditions of Fischer‐Tropsch Synthesis,” Chemical Communications 46 (2010): 788–790, 10.1039/B920110F. 20087521

[19] K. F. Tan , J. Xu , J. Chang , A. Borgna , and M. Saeys , “Carbon Deposition on Co Catalysts During Fischer–Tropsch Synthesis: A Computational and Experimental Study,” Journal of Catalysis 274 (2010): 121–129, 10.1016/j.jcat.2010.06.008.

[20] M. Claeys , M. E. Dry , E. van Steen , et al., “In Situ Magnetometer Study on the Formation and Stability of Cobalt Carbide in Fischer–Tropsch Synthesis,” Journal of Catalysis 318 (2014): 193–202, 10.1016/j.jcat.2014.08.002.

[21] J. G. Moya‐Cancino , A.‐P. Honkanen , A. M. J. van der Eerden , et al., “In Situ X‐ray Raman Scattering Spectroscopy of the Formation of Cobalt Carbides in a Co/TiO_2_ Fischer–Tropsch Synthesis Catalyst,” ACS Catalysis 11 (2021): 809–819, 10.1021/acscatal.0c04509.

[22] P. Hazemann , D. Decottignies , S. Maury , S. Humbert , F. C. Meunier , and Y. Schuurman , “Selectivity Loss in Fischer‐Tropsch Synthesis: The Effect of Cobalt Carbide Formation,” Journal of Catalysis 397 (2021): 1–12, 10.1016/j.jcat.2021.03.005.

[23] P. Lömker , D. Degerman , C. M. Goodwin , et al., “In‐situ Probing of the Fischer‐Tropsch Reaction on Co Single Crystal Surfaces up to 1 bar,” Nature Communications 16 (2025): 1005, 10.1038/s41467-025-56082-8. PMC11761050 39856064

[24] J. Wilson and C. de Groot , “Atomic‐Scale Restructuring in High‐Pressure Catalysis,” Journal of Physical Chemistry 99 (1995): 7860–7866, 10.1021/j100020a005.

[25] H. Schulz , Z. Nie , and F. Ousmanov , “Construction of the Fischer–Tropsch Regime With Cobalt Catalysts,” Catalysis Today 71 (2002): 351–360, 10.1016/S0920-5861(01)00462-X.

[26] A. Banerjee , V. Navarro , J. W. M. Frenken , A. P. van Bavel , H. P. C. E. Kuipers , and M. Saeys , “Shape and Size of Cobalt Nanoislands Formed Spontaneously on Cobalt Terraces During Fischer–Tropsch Synthesis,” Journal of Physical Chemistry Letters 7 (2016): 1996–2001, 10.1021/acs.jpclett.6b00555. 27176712

[27] X.‐Q. Zhang , R. A. van Santen , and E. J. M. Hensen , “Carbon‐Induced Surface Transformations of Cobalt,” ACS Catalysis 5 (2015): 596–601, 10.1021/cs501484c.

[28] M. Salmeron and B. Eren , “High‐Pressure Scanning Tunneling Microscopy,” Chemical Reviews 121 (2021): 962–1006, 10.1021/acs.chemrev.0c00429. 33290057

[29] J. Schnadt , J. Knudsen , and N. Johansson , “Present and New Frontiers in Materials Research by Ambient Pressure X‐ray Photoelectron Spectroscopy,” Journal of Physics‐Condensed Matter 32 (2020): 413003, 10.1088/1361-648x/ab9565. 32438360

[30] F. Zaera , “New Advances in the Use of Infrared Absorption Spectroscopy for the Characterization of Heterogeneous Catalytic Reactions,” Chemical Society Reviews 43 (2014): 7624–7663, 10.1039/C3CS60374A. 24424375

[31] C. J. Weststrate , A. C. Kızılkaya , E. T. R. Rossen , et al., “Atomic and Polymeric Carbon on Co(0001): Surface Reconstruction, Graphene Formation, and Catalyst Poisoning,” Journal of Physical Chemistry C 116 (2012): 11575–11583, 10.1021/jp301706q.

[32] L. Xu , Y. Ma , Z. Wu , B. Chen , Q. Yuan , and W. Huang , “A Photoemission Study of Ethylene Decomposition on a Co(0001) Surface: Formation of Different Types of Carbon Species,” Journal of Physical Chemistry C 116 (2012): 4167–4174, 10.1021/jp210500p.

[33] J. Nakamura , I. Toyoshima , and K. I. Tanaka , “Formation of Carbidic and Graphite Carbon From CO on Polycrystalline Cobalt,” Surface Science 201 (1988): 185–194, 10.1016/0039-6028(88)90605-X.

[34] B. Böller , M. Ehrensperger , and J. Wintterlin , “In Situ Scanning Tunneling Microscopy of the Dissociation of CO on Co(0001),” ACS Catalysis 5 (2015): 6802–6806, 10.1021/acscatal.5b01684.

[35] J. J. McCarroll , T. Edmonds , and R. C. Pitkethly , “Interpretation of a Complex Low Energy Electron Diffraction Pattern: Carbonaceous and Sulphur‐Containing Structures on Ni(111),” Nature 223 (1969): 1260–1262, 10.1038/2231260a0.

[36] C. Klink , I. Stensgaard , F. Besenbacher , and E. Lægsgaard , “An STM Study of Carbon‐Induced Structures on Ni(111): Evidence for a Carbidic‐phase Clock Reconstruction,” Surface Science 342 (1995): 250–260, 10.1016/0039-6028(95)00697-4.

[37] C. J. Weststrate , P. van Helden , J. van de Loosdrecht , and J. W. Niemantsverdriet , “Elementary Steps in Fischer–Tropsch Synthesis: CO Bond Scission, CO Oxidation and Surface Carbiding on Co(0001),” Surface Science 648 (2016): 60–66, 10.1016/j.susc.2015.10.050.

[38] C. J. Weststrate , H. J. Gericke , M. W. G. M. Verhoeven , I. M. Ciobîcă , A. M. Saib , and J. W. Niemantsverdriet , “Ethanol Decomposition on Co(0001): C−O Bond Scission on a Close‐Packed Cobalt Surface,” Journal of Physical Chemistry Letters 1 (2010): 1767–1770, 10.1021/jz100537h.

[39] J. Cheng , P. Hu , P. Ellis , S. French , G. Kelly , and C. M. Lok , “Density Functional Theory Study of Iron and Cobalt Carbides for Fischer−Tropsch Synthesis,” Journal of Physical Chemistry C 114 (2010): 1085–1093, 10.1021/jp908482q.

[40] J. C. Mohandas , M. K. Gnanamani , G. Jacobs , et al., “Fischer–Tropsch Synthesis: Characterization and Reaction Testing of Cobalt Carbide,” ACS Catalysis 1 (2011): 1581–1588, 10.1021/cs200236q.

[41] B. Zijlstra , R. J. P. Broos , W. Chen , G. L. Bezemer , I. A. W. Filot , and E. J. M. Hensen , “The Vital Role of Step‐Edge Sites for both CO Activation and Chain Growth on Cobalt Fischer–Tropsch Catalysts Revealed Through First‐Principles‐Based Microkinetic Modeling Including Lateral Interactions,” ACS Catalysis 10 (2020): 9376–9400, 10.1021/acscatal.0c02420.

[42] K. T. Rommens , G. T. K. K. Gunasooriya , and M. Saeys , “Key Role of CO Coverage for Chain Growth in Co‐Based Fischer–Tropsch Synthesis,” ACS Catalysis 14 (2024): 6696–6709, 10.1021/acscatal.3c04844.

[43] C. J. Weststrate , D. Sharma , D. G. Rodríguez , H. O. A. Fredriksson , and J. W. Niemantsverdriet , “Water Formation Kinetics on Co(0001) at Low and Near‐Ambient Hydrogen Pressures in the Context of Fischer–Tropsch Synthesis,” Journal of Physical Chemistry C 127 (2023): 3452–3461, 10.1021/acs.jpcc.2c08092.

[44] B. G. Johnson , C. H. Bartholomew , and D. W. Goodman , “The Role of Surface Structure and Dispersion in CO Hydrogenation on Cobalt,” Journal of Catalysis 128 (1991): 231–247, 10.1016/0021-9517(91)90080-N.

[45] P. van Helden , I. M. Ciobîcă , and R. L. J. Coetzer , “The Size‐dependent Site Composition of FCC Cobalt Nanocrystals,” Catalysis Today 261 (2016): 48–59, 10.1016/j.cattod.2015.07.052.

[46] G. Melaet , W. T. Ralston , C.‐S. Li , et al., “Evidence of Highly Active Cobalt Oxide Catalyst for the Fischer–Tropsch Synthesis and CO_2_ Hydrogenation,” Journal of the American Chemical Society 136 (2014): 2260–2263, 10.1021/ja412447q. 24460136

[47] L. Zhong , F. Yu , Y. An , et al., “Cobalt Carbide Nanoprisms for Direct Production of Lower Olefins From Syngas,” Nature 538 (2016): 84–87, 10.1038/nature19786. 27708303

[48] I. K. van Ravenhorst , A. S. Hoffman , C. Vogt , et al., “On the Cobalt Carbide Formation in a Co/TiO_2_ Fischer–Tropsch Synthesis Catalyst as Studied by High‐Pressure, Long‐Term *Operando* X‐ray Absorption and Diffraction,” ACS Catalysis 11 (2021): 2956–2967, 10.1021/acscatal.0c04695. 33815895 PMC8016113

[49] Y.‐P. Pei , J.‐X. Liu , Y.‐H. Zhao , et al., “High Alcohols Synthesis via Fischer–Tropsch Reaction at Cobalt Metal/Carbide Interface,” ACS Catalysis 5 (2015): 3620–3624, 10.1021/acscatal.5b00791.

[50] G. Prieto , M. I. S. De Mello , P. Concepción , R. Murciano , S. B. C. Pergher , and A. Martínez , “Cobalt‐Catalyzed Fischer–Tropsch Synthesis: Chemical Nature of the Oxide Support as a Performance Descriptor,” ACS Catalysis 5 (2015): 3323–3335, 10.1021/acscatal.5b00057.

[51] N. E. Tsakoumis , A. Voronov , M. Rønning , et al., “Fischer–Tropsch Synthesis: An XAS/XRPD Combined In Situ Study From Catalyst Activation to Deactivation,” Journal of Catalysis 291 (2012): 138–148, 10.1016/j.jcat.2012.04.018.

[52] K. Høydalsvik , J. B. Fløystad , A. Voronov , et al., “Morphology Changes of Co Catalyst Nanoparticles at the Onset of Fischer–Tropsch Synthesis,” Journal of Physical Chemistry C 118 (2014): 2399–2407, 10.1021/jp4052193.

[53] M. Rößler , P. Geng , and J. Wintterlin , “A High‐pressure Scanning Tunneling Microscope for Studying Heterogeneous Catalysis,” Review of Scientific Instruments 76 (2005): 023705, 10.1063/1.1841951.

[54] K. M. Golder , B. Böller , G. Stienen , J. Sickerling , and J. Wintterlin , “A Highly Sensitive Gas Chromatograph for In Situ and *Operando* Experiments on Catalytic Reactions,” Review of Scientific Instruments 92 (2021): 124103, 10.1063/5.0068021. 34972407

[55] G. Kresse and J. Furthmüller , “Efficient Iterative Schemes for Ab Initio Total‐Energy Calculations Using a Plane‐wave Basis Set,” Physical Review B 54 (1996): 11169–11186, 10.1103/PhysRevB.54.11169. 9984901

[56] P. E. Blöchl , “Projector Augmented‐wave Method,” Physical Review B 50 (1994): 17953–17979, 10.1103/PhysRevB.50.17953. 9976227

[57] B. Hammer , L. B. Hansen , and J. K. Nørskov , “Improved Adsorption Energetics Within Density‐functional Theory Using Revised Perdew‐Burke‐Ernzerhof Functionals,” Physical Review B 59 (1999): 7413–7421, 10.1103/PhysRevB.59.7413.

[58] S. Grimme , J. Antony , S. Ehrlich , and H. Krieg , “A Consistent and Accurate Ab Initio Parametrization of Density Functional Dispersion Correction (DFT‐D) for the 94 Elements H‐Pu,” Journal of Chemical Physics 132 (2010): 154104, 10.1063/1.3382344. 20423165

[59] S. Grimme , “Density Functional Theory With London Dispersion Corrections,” Wiley Interdisciplinary Reviews: Computational Molecular Science 1 (2011): 211–228, 10.1002/wcms.30.

[60] S. Grimme , A. Hansen , J. G. Brandenburg , and C. Bannwarth , “Dispersion‐Corrected Mean‐Field Electronic Structure Methods,” Chemical Reviews 116 (2016): 5105–5154, 10.1021/acs.chemrev.5b00533. 27077966

[61] D. Mahlberg , S. Sakong , K. Forster‐Tonigold , and A. Groß , “Improved DFT Adsorption Energies With Semiempirical Dispersion Corrections,” Journal of Chemical Theory and Computation 15 (2019): 3250–3259, 10.1021/acs.jctc.9b00035. 30964999

[62] M. W. Chase Jr. , “NIST‐JANAF Thermochemical Tables,” Journal of Physical and Chemical Reference Data, Monograph 9 (1998): 1–1951.

[63] C. J. Weststrate , I. M. Ciobîcă , A. M. Saib , D. J. Moodley , and J. W. Niemantsverdriet , “Fundamental Issues on Practical Fischer–Tropsch Catalysts: How Surface Science Can Help,” Catalysis Today 228 (2014): 106–112, 10.1016/j.cattod.2013.11.

