# Supplementary material for: A Carbon‐Induced Surface Reconstruction on an Operating Fischer–Tropsch Catalyst
Source: Angew Chem Int Ed Engl. 2026 Jul 16;65(38):e5104870. doi: 10.1002/anie.5104870 (PMC13573073; doi:10.1002/anie.5104870)
Supplement: Supplementary file 1 [file ANIE-65-e5104870-s001.pdf]

Supporting Information

A Carbon-Induced Surface Reconstruction on an Operating Fischer–Tropsch Catalyst

Sebastian Kläger[1], Sung Sakong[2], Axel Groß[2], and Joost Wintterlin[1,3]*

[1]Department of Chemistry, Ludwig-Maximilians-Universität München, Butenandtstr. 5–13, 81377 Munich, Germany

[2]Institute of Theoretical Chemistry, University of Ulm, Oberberghof 7, 89081 Ulm, Germany

[3]Center for NanoScience, Schellingstr. 4, 80799 Munich, Germany

*Corresponding Author; E-Mail: wintterlin@cup.uni-muenchen.de

Table of Contents

A. Experimental Section	3
B. Computational Methods	5
C. Supporting Figures and Tables	6
References	13

A. Experimental Section

Apparatus. The setup used for the experiments consists of a UHV chamber and an attached reaction cell with GC. The UHV chamber is equipped with an XP spectrometer, an ion gun, low-energy electron diffraction (LEED) optics, a quadrupole mass spectrometer, and a sample manipulator. The reaction cell (volume 1.8 L) houses the STM and is connected to the UHV chamber by a sample transfer system. The cell is coated with amorphous Si (SilcoGuard 1000, SilcoTek) to prevent Ni carbonyl formation at the stainless-steel walls. Experiments in the cell can be performed between UHV and ~1 bar; sample heating is accomplished by an infrared laser connected to the sample holder of the STM setup by a glass fiber. For details, we refer to a previous publication.[1]

STM. The beetle-type STM is home-built. Pt/Ir tips were used; tunneling voltages and currents are given in the respective figure captions. For scan control and image recording, home-developed software was applied. For image processing (plane correction, line noise reduction, contrast adjustment, minor Gaussian filtering), Igor Pro software (WaveMetrics) was used.

XPS. The XP spectrometer consists of a monochromatized Al Kα X-ray source (XM 1000 MkII, Omicron) and a hemispherical analyzer (PHOIBOS 100 MCD5, SPECS). Peaks were fitted with Gaussians convoluted with Doniach-Šunjić functions after subtracting linear backgrounds. CO and carbon coverages were determined by applying the respective cross sections and asymmetry parameters of the O 1s and C 1s orbitals and normalizing by the Co $2p_{1/2}$ peak intensity (taking into account the exponentially decaying contributions from deeper Co layers to this peak).

GC. The GC is configured to analyze extremely low concentrations of hydrocarbons. It consists of a basic unit (Trace 1310, Thermo Scientific), equipped with a 2 m HayeSep Q80/100 micropacked column (Restek) plus a 30 m TracePLOT TG-BOND Q+ column (Thermo Scientific), a flame ionization detector, and a special gas sampling unit (S+H Analytik). Utilizing a cold trap at the column head for gas concentration and peak focusing, a detection limit of 0.4 ppb is achieved. The design has been described in detail before.[2] Calibration was performed with a custom-made mixture of C_1 to C_4 hydrocarbons. TOFs of CH_4 and the saturated and unsaturated C_2 to C_4 hydrocarbons were determined per Co surface atom from the increments of the respective signals per hour.

Sample. A round Co(0001) single crystal (MaTecK) with a polished surface of 5 mm in diameter (surface roughness lower than 0.03 µm, orientation better than 0.1°) was used as a sample. The surface was prepared in the UHV chamber by repeated Ne^+ sputtering (1 keV, 10 min) and annealing at 623 K (well below the hcp-to-fcc phase transition of Co at ~700 K), until the C 1s signal was at the XPS detection limit (carbon was the primary contaminant).

Syngas. The reactor cell was filled with syngas by sequentially introducing CO (99.99%, Air Liquide) and H_2 (99.9999%, Air Liquide) in a 1:2 ratio. The total pressure was 950 mbar, avoiding overpressure. H_2 was cleaned using purifiers (MC1-904, MC50-403, SAES Pure Gas) and a liquid N_2 cold trap. CO was cleaned, mainly to remove Ni carbonyls, by a hot trap (a Cu pipe filled with molecular sieve at 300 °C) and a liquid N_2 cold trap. A low, variable Ni signal, detected by XPS after 6 h reaction time, was not correlated with the activity and does not seem to play a significant role in the reaction.

Experimental procedure. After surface cleaning in the UHV chamber, a carbide layer was prepared as described in the main text. The sample was then transferred to the reactor cell and, in most cases, first investigated by STM in UHV at room temperature. Then, syngas was introduced, and the heating laser was turned on. When the temperature had stabilized (usually after 20 min), STM recording was started or, alternatively, gas samples were extracted by the GC sampling unit (once per hour). Although possible in principle, these two experiments were not performed simultaneously to simplify the

practical conduct of the measurements. Because the conditions are identical, we can still speak of *operando* experiments. Reaction experiments lasted several hours, up to 6 h. Despite the batch mode, the amount of syngas did not change significantly due to the low conversions and the low gas volumes extracted for GC. Differential conditions were thus maintained. At the end of an experiment, the heating laser was turned off, and when the sample had cooled to ∼70 °C in syngas, the reactor cell was evacuated. Finally, the sample was transferred to the UHV chamber for analysis by XPS. In the comparative experiments with the metallic surface, this entire procedure was performed with the sample as obtained from the cleaning procedure in UHV.

B. Computational Methods

Spin-polarized DFT calculations were performed using the periodic density functional theory package VASP.[3] The carbon structures of the Co(0001) surface were modeled by slabs of five atomic layers with Co vacancies and C atoms on the surface. Total energies of slab configurations were determined by electron wave functions expanded with a cutoff energy of 450 eV using a plane-wave basis set. The electronic atom cores were described by the projector augmented wave (PAW) method,[4] and the exchange-correlation energies were evaluated within the generalized gradient approximation suggested by Hammer and Nørskov, a revised version of the Perdew-Burke-Ernzerhof (RPBE) functional.[5] Dispersion effects between the top Co layer and adsorbates were treated within Grimme's semi-empirical D3 dispersion correction scheme.[6-9] Energies were determined within (3×3), (4×4), (5×5), and (7×7) surface supercells employing (5×5), (4×4), (3×3), and (2×2) k-point grids in the first Brillouin zone, respectively. The computed energies satisfy the convergence criterion of 10^{-6} eV. The Co atoms in the two bottom layers were fixed at their bulk positions, while the Co atoms in the three top layers and the C atoms were fully relaxed with a force convergence criterion of 0.01 eV. The bulk lattice parameters of hexagonal Co (a = 2.51 Å and c/a = 1.61) were obtained by calculations with a dense k-point mesh of $(25 \times 25 \times 25)$.

The formation energy of an adsorbed C atom, $E_\mathrm{f} = \Delta E / n_\mathrm{C}$, in a given structural configuration was computed as an energy difference $\Delta E = E_\mathrm{tot} - E_\mathrm{Co(0001)} + n_\mathrm{V} E_\mathrm{Co(b)} - n_\mathrm{C} E_\mathrm{C}$. E_tot, $E_\mathrm{Co(0001)}$, and $E_\mathrm{Co(b)}$ are the energies of slabs with adsorbed C atoms and Co vacancies, of slabs of the bare Co(0001) surface, and of bulk Co atoms, respectively. (Vacancies were included because the reconstruction model has fewer Co atoms than a close-packed layer.) n_V and n_C are the numbers of Co vacancies and C atoms, respectively. Note that the Co vacancies considered here are not related to the mesoscopic surface holes resulting from the reduced Co atom density after carbide decomposition. The energy E_C is defined by gas-phase CO, H_2O, and H_2 energies, i.e., $E_\mathrm{C} = E_\mathrm{CO(g)} - E_\mathrm{H_2O(g)} + E_\mathrm{H_2(g)}$. In effect, E_f represents the energy of a C atom in a certain structural configuration relative to the flat, bare Co(0001) surface and to the CO(g) + H_2(g) ⇌ C + H_2O(g) equilibrium.

As suggested by Banerjee et al.,[10] carbon chemical potentials were obtained from the same equilibrium as follows:

$$\mu_\mathrm{C}(T,p) = \mu_\mathrm{CO}(T,p) + \mu_\mathrm{H_2}(T,p) - \mu_\mathrm{H_2O}(T,p) = \Delta H - T\Delta S + \mathrm{R}T \ln(p_\mathrm{CO} p_\mathrm{H_2} / p_\mathrm{H_2O})$$

The enthalpy change ΔH was evaluated from DFT values, while the entropy change ΔS was estimated using experimental values of gas molecules.[11]

C. Supporting Figures and Tables

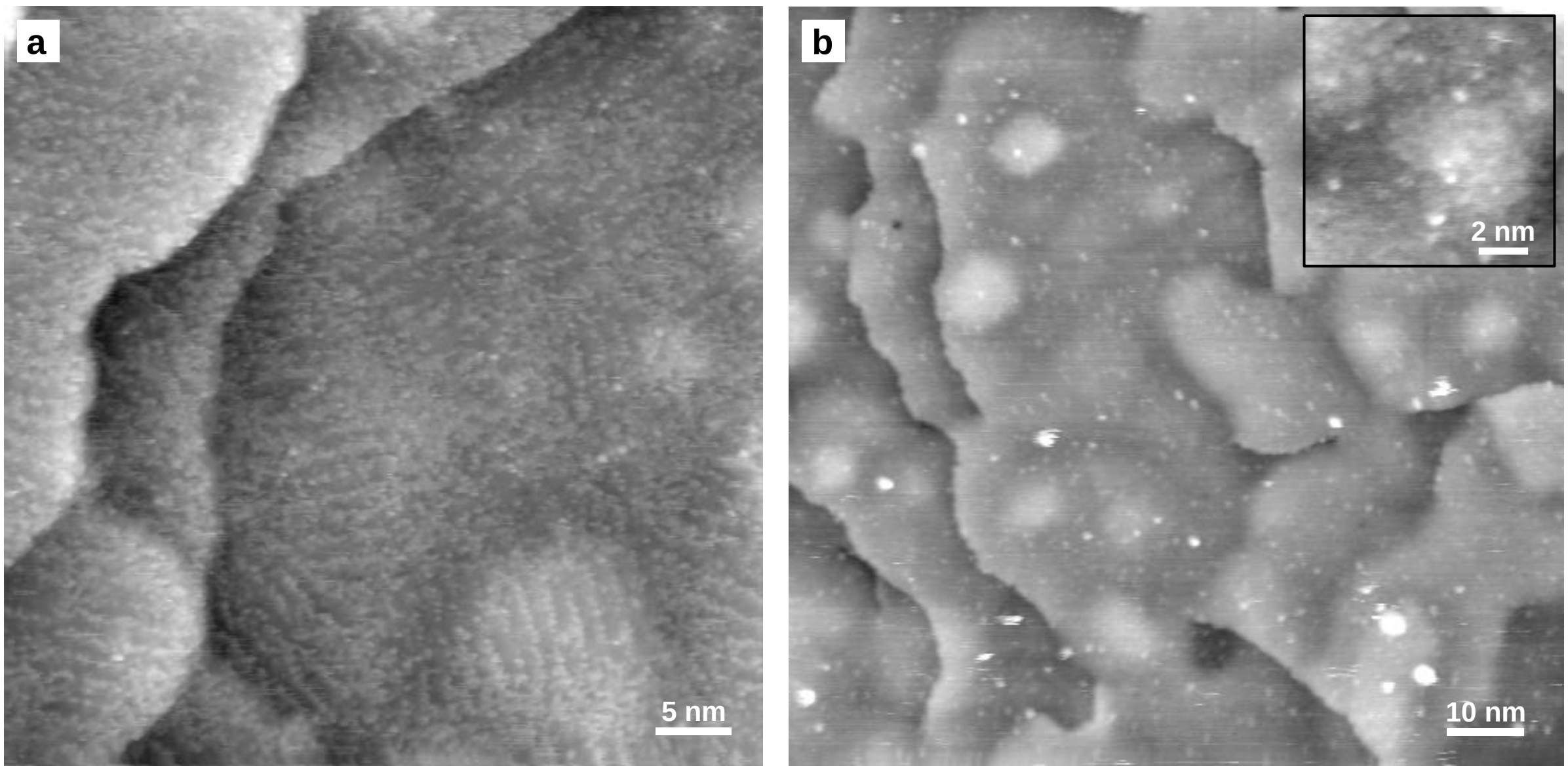

Figure S1. STM images of the carbide-covered Co(0001) surface under syngas at room temperature. (a) Image recorded after 5 h in ~1 × 10^{-8} mbar syngas (containing a low amount of H_2O), V_t = −0.3 V, I_t = 2.3 nA. The stripe structure of the carbide (Fig. 2b) has started to become disordered. (b) Images recorded in 950 mbar syngas, immediately after filling the STM cell, V_t = −0.2 V, I_t = 2.3 nA. The stripe pattern has completely disappeared, and the surface is covered by an amorphous layer. Because the temperature has not yet been increased to the reaction regime, the reconstruction has not yet formed.

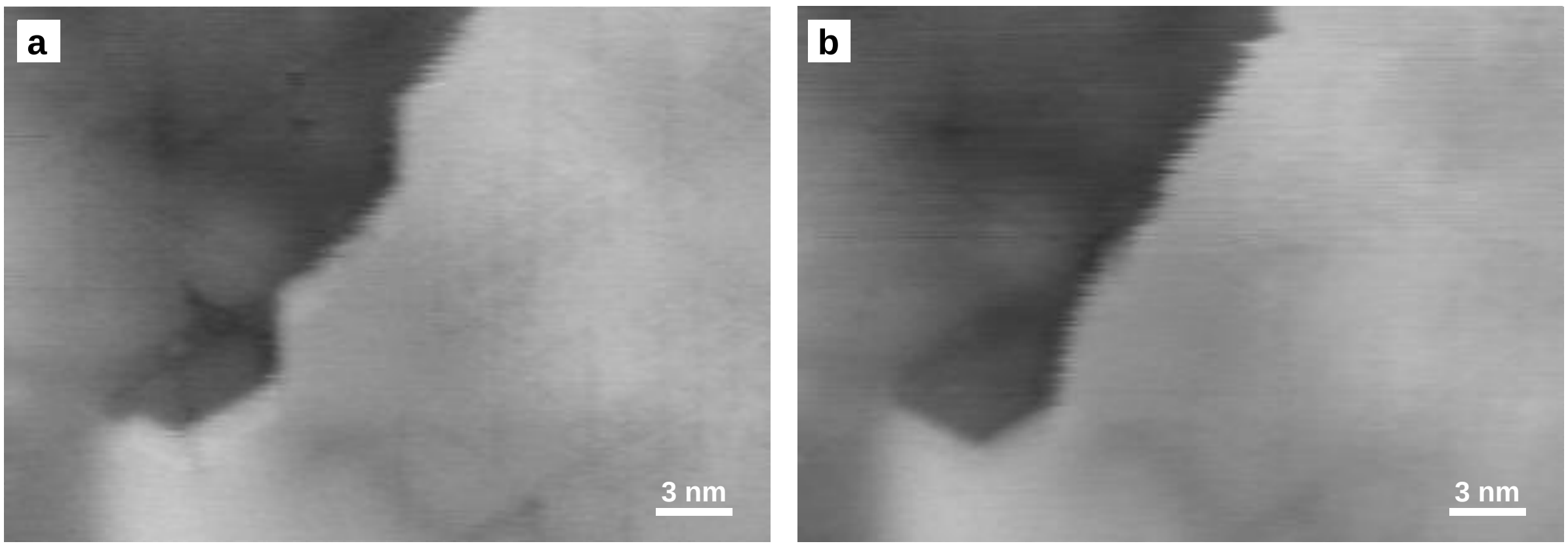

Figure S2. STM images showing the changing orientation of a step edge over time under reaction conditions (950 mbar syngas, 473 K). (a) Image recorded 2:15 h and (b) image recorded 2:45 h after starting the reaction, V_t = –0.2 V, I_t = 2.3 nA. After disintegration of the carbide, many steps show straight segments (a), but over time, these segments disappear, and the steps adopt the irregular, fringed shape typical of the experiments with the metallic surface (b). When not pinned by a strongly bound adsorbate, Co step atoms rapidly fluctuate, leading to a fringed appearance of steps in the STM.[12] The straight parts can be explained by residual C atoms bound at the steps and stabilizing a certain geometry (as has been shown by DFT).[13] In the course of the reaction, these C atoms are reacted away.

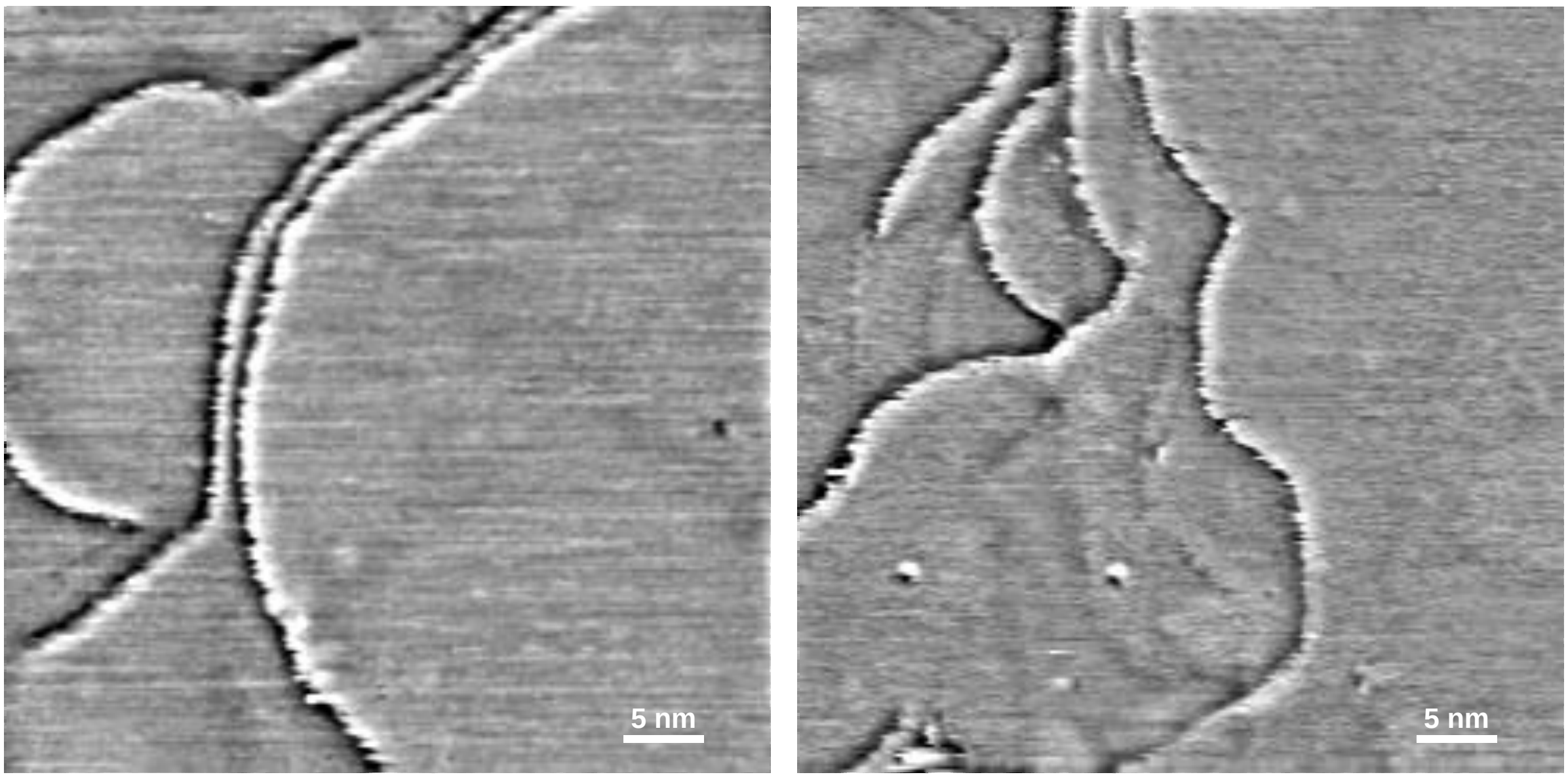

Figure S3. STM images of the originally bare Co(0001) sample under reaction conditions (950 mbar syngas, 473 K), V_t = −0.5 V, I_t = 0.7 nA. The contrast is enhanced in the same way as in Fig. 4 of the main text, but in contrast to the originally carbide-covered surface, there is no indication of the triangle reconstruction on the terraces.

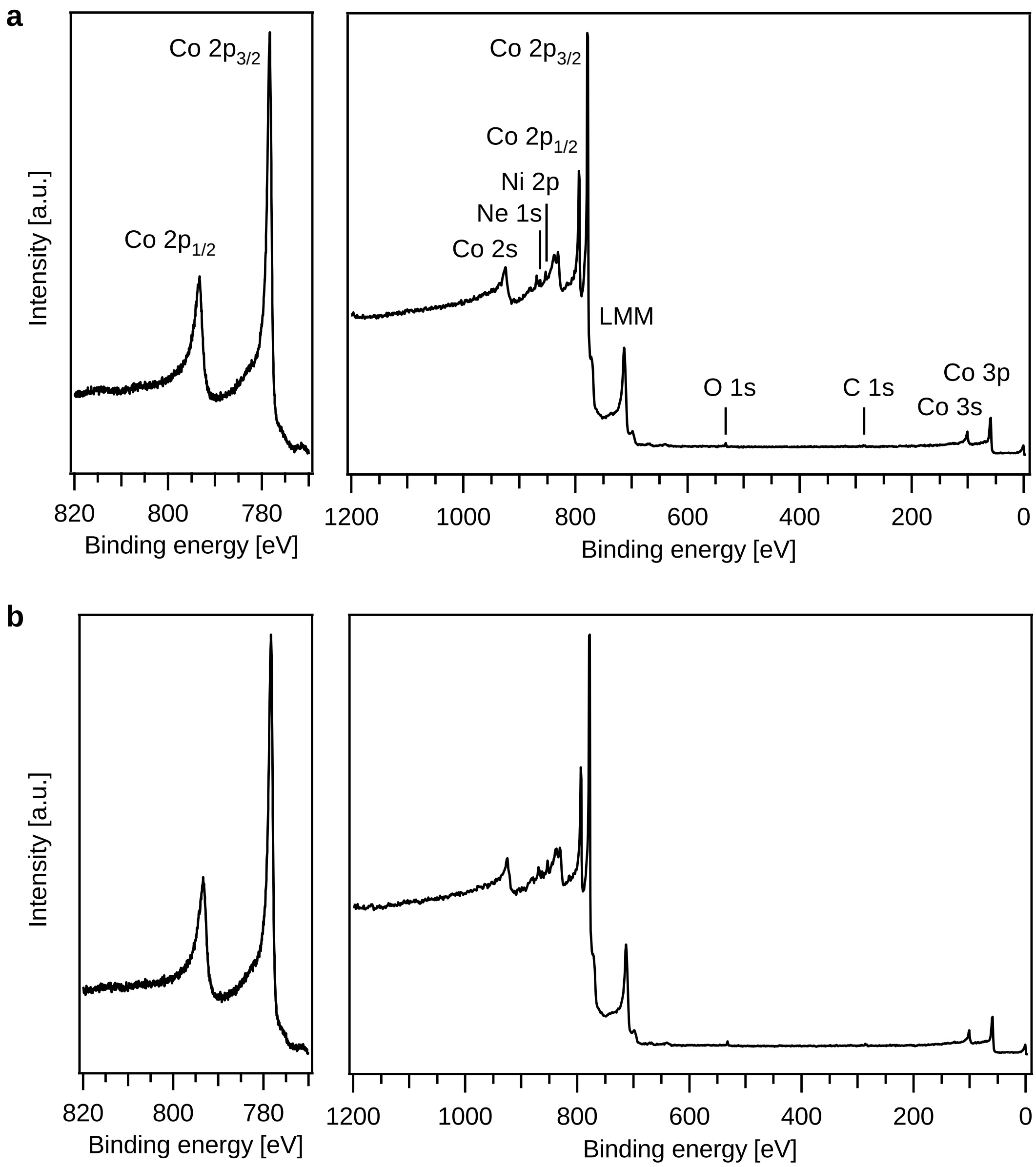

Figure S4. XP spectra of the Co 2p region and survey spectra of the Co(0001) surface. (a) Spectra taken after exposure of the bare surface to syngas for 4 h (473 K, 950 mbar). (b) Spectra taken after exposure of the carbide-covered surface to the same reaction conditions. There are no measurable differences between the spectra in the two experiments.

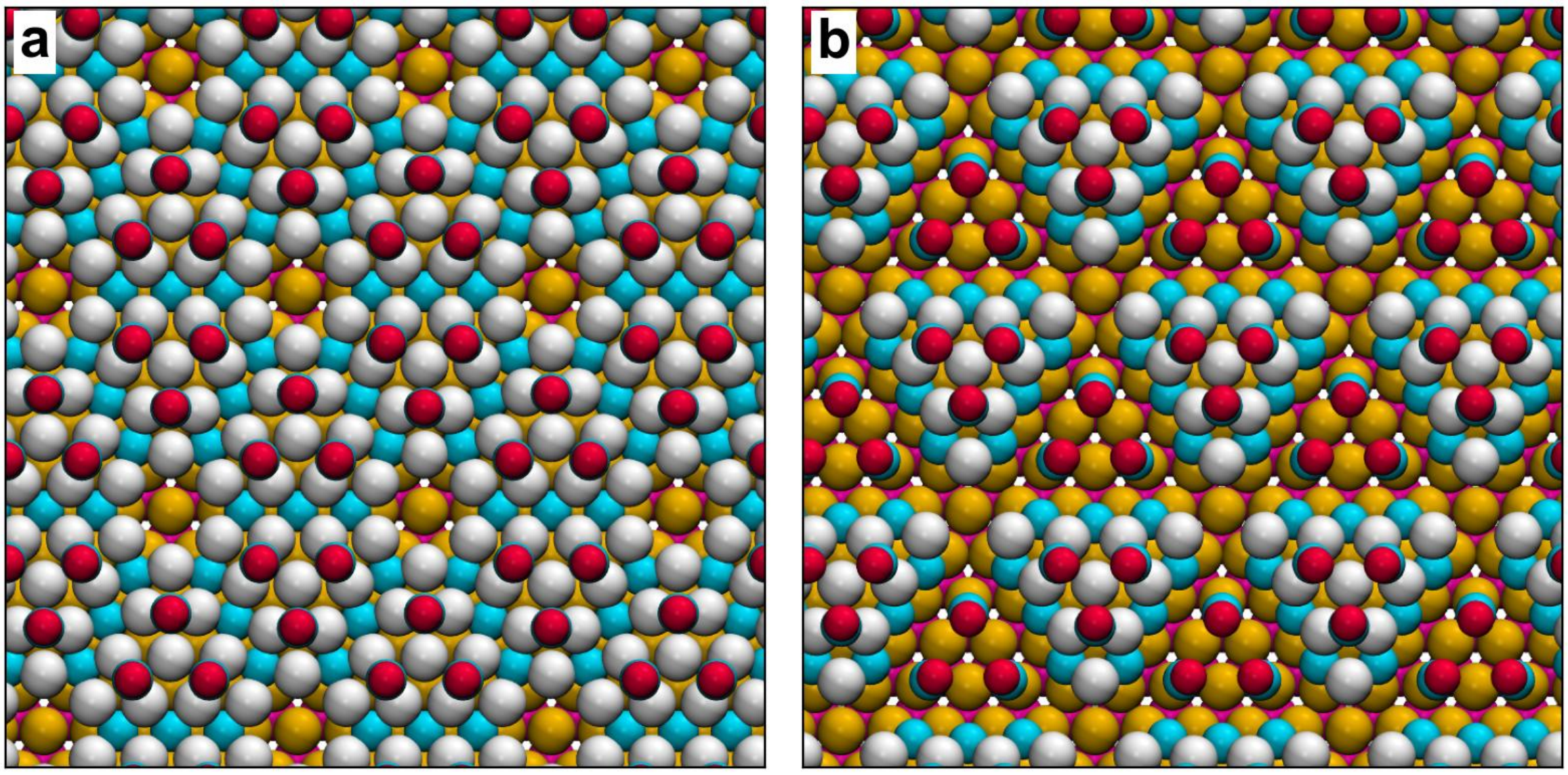

Figure S5. (a) Optimized C-induced triangular stacking-fault reconstruction and (b) C-saturated triangular island with (5×5) unit cells on the Co(0001) surface with six adsorbed CO molecules per unit cell as obtained by DFT. The CO adsorption energies are 1.76 and 1.70 eV/CO, respectively. These values are higher than the adsorption energy of an isolated CO molecule (1.64 eV) on a flat surface at a coverage of 0.04 ML, indicating that CO adsorption stabilizes both surfaces by approximately the same degree. Cyan and red balls represent C and O atoms, and white, yellow, and pink balls are Co atoms in the first, second, and third layers, respectively.

a

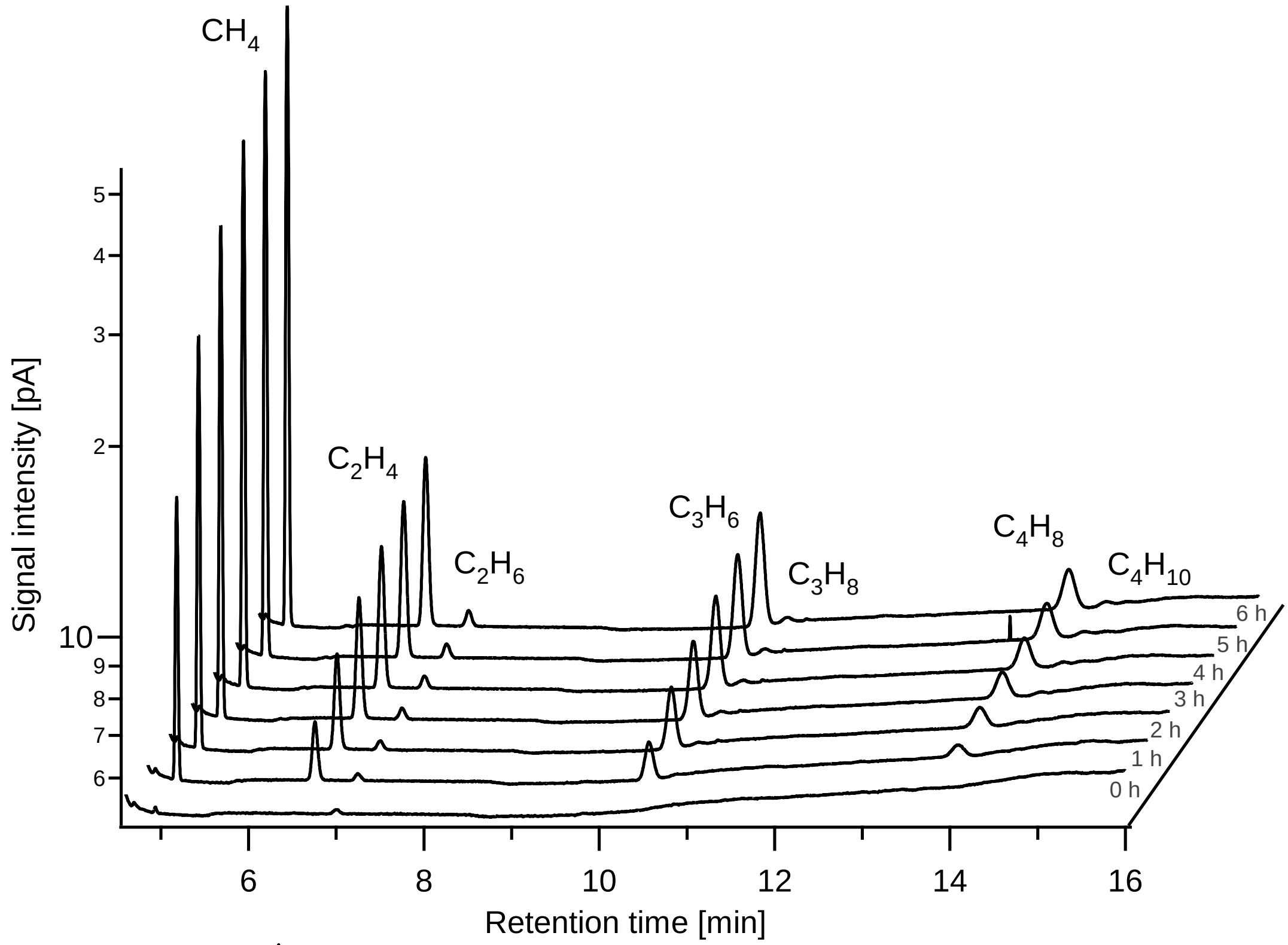

b

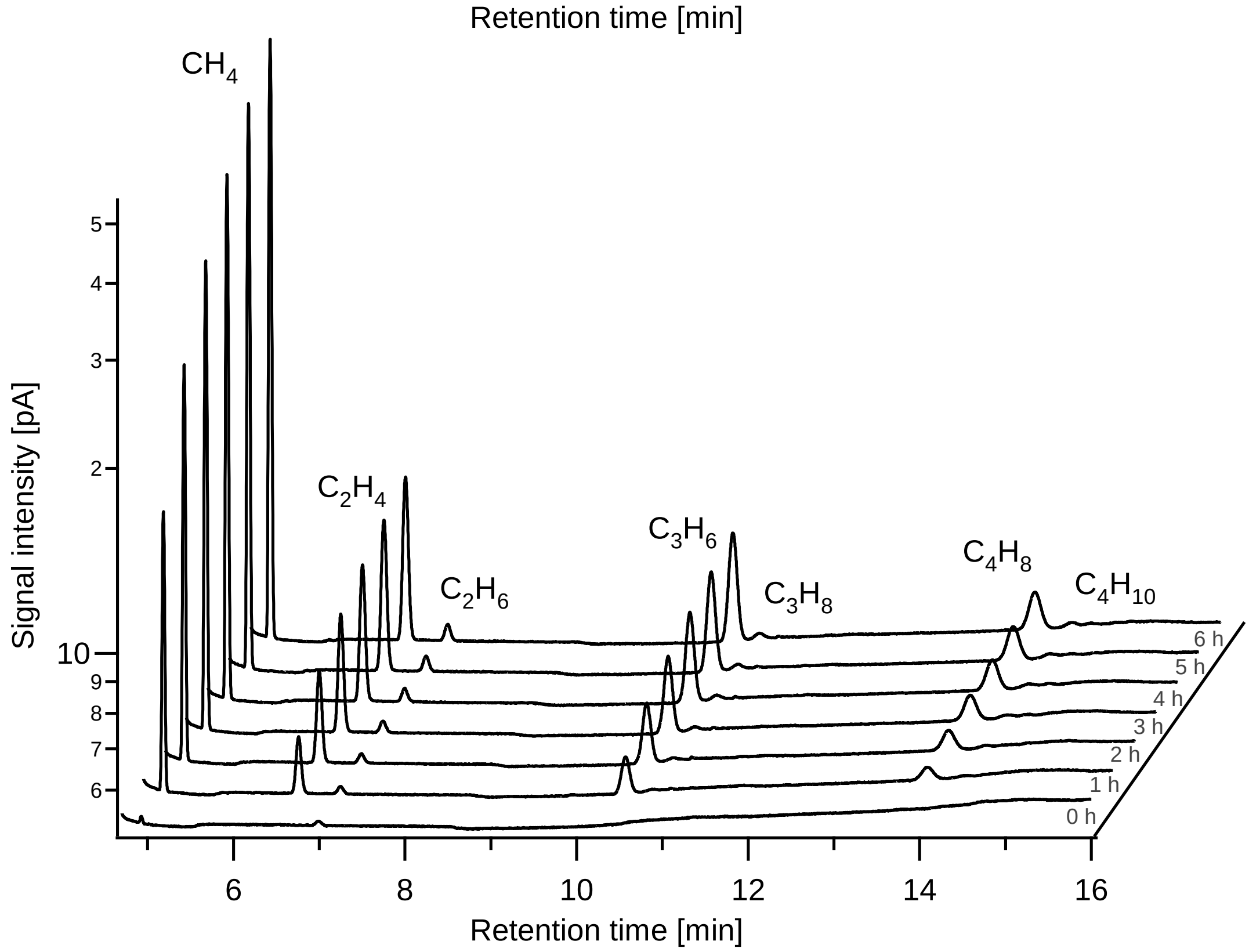

Figure S6. Gas chromatograms taken during 6 h reaction experiments with a Co(0001) sample. In (a), the sample was initially in a metallic state, and in (b), the sample was initially covered with surface carbide. Chromatograms were taken every hour; reaction conditions were 950 mbar of syngas and a sample temperature of 493 K.

Table S1. Turnover frequencies (TOFs) of the individual products and of the consumption of CO from reaction experiments (950 mbar syngas, H_2:CO = 2:1) with an initially bare Co(0001) sample

	TOF(CH_4) [10^{-3} s^{-1}]	TOF(C_2H_4) [10^{-3} s^{-1}]	TOF(C_2H_6) [10^{-3} s^{-1}]	TOF(C_3H_6) [10^{-3} s^{-1}]	TOF(C_3H_8) [10^{-3} s^{-1}]	TOF(C_4H_8) [10^{-3} s^{-1}]	TOF(C_4H_{10}) [10^{-3} s^{-1}]	TOF(CO) [10^{-3} s^{-1}]
Experiment A (493 K)‡								
2 h	6.8	0.84	0.044	0.50	0.020	0.20	0.017	10.9
3 h	5.6	0.62	0.043	0.43	0.033	0.19	0.013	9.1
4 h	4.6	0.47	0.033	0.36	0.010	0.13	0.020	7.3
5 h	4.0	0.43	0.043	0.35	0.013	0.15	0.020	6.7
6 h	3.4	0.35	0.026	0.27	0.013	0.11	0.013	5.4
Experiment B (493 K)‡								
2 h	6.9	0.87	0.054	0.55	0.023	0.23	0.010	11.4
3 h	6.0	0.66	0.033	0.41	0.014	0.14	0.020	9.3
4 h	5.3	0.53	0.027	0.37	0.015	0.15	0.017	8.2
5 h	4.5	0.44	0.043	0.30	0.013	0.13	0.016	7.1
6 h	4.1	0.38	0.029	0.28	0.010	0.10	0.010	6.3
Experiment C (493 K)‡								
2 h	7.1	0.92	0.040	0.58	0.017	0.20	0.013	11.6
3 h	6.0	0.65	0.043	0.40	0.016	0.14	0.013	9.2
4 h	5.6	0.56	0.024	0.36	0.010	0.13	0.014	8.4
5 h	4.7	0.44	0.040	0.28	0.010	0.14	0.016	7.2
6 h	4.3	0.41	0.029	0.30	0.013	0.10	0.010	6.5
Experiment D (473 K)								
1 h	2.7	0.49	0.020	0.38	0.020	0.17	-*	5.6
2 h	2.4	0.40	0.020	0.30	0.020	0.13	-*	4.7
3 h	2.2	0.33	0.013	0.26	0.010	0.081	-*	4.0
4 h	2.0	0.28	0.027	0.19	0.010	0.081	-*	3.5

‡ 1 h TOFs discarded because of slow temperature adjustment

* TOF below quantification limit

Table S2. TOFs of the individual products and of the consumption of CO from reaction experiments (950 mbar syngas, H_2:CO = 2:1) with an initially carbide-covered Co(0001) sample

	TOF(CH_4) [10^{-3} s^{-1}]	TOF(C_2H_4) [10^{-3} s^{-1}]	TOF(C_2H_6) [10^{-3} s^{-1}]	TOF(C_3H_6) [10^{-3} s^{-1}]	TOF(C_3H_8) [10^{-3} s^{-1}]	TOF(C_4H_8) [10^{-3} s^{-1}]	TOF(C_4H_{10}) [10^{-3} s^{-1}]	TOF(CO) [10^{-3} s^{-1}]
Experiment A (493 K)‡								
2 h	6.7	0.88	0.044	0.57	0.023	0.20	0.013	11.2
3 h	5.8	0.69	0.050	0.43	0.020	0.14	0.013	9.3
4 h	5.4	0.58	0.030	0.35	0.013	0.12	0.013	8.2
5 h	4.7	0.48	0.049	0.29	0.013	0.12	0.010	7.2
6 h	4.4	0.41	0.023	0.28	0.013	0.089	0.007	6.5
Experiment B (493 K)‡								
2 h	6.5	0.88	0.047	0.59	0.013	0.19	0.007	10.9
3 h	5.8	0.70	0.037	0.41	0.023	0.15	0.023	9.2
4 h	5.4	0.59	0.050	0.35	0.013	0.12	0.007	8.2
5 h	4.7	0.48	0.036	0.28	0.007	0.12	0.016	7.2
6 h	4.5	0.43	0.033	0.29	0.013	0.088	0.010	6.7
Experiment C (493 K)‡								
2 h	6.8	0.91	0.043	0.57	0.013	0.21	0.010	11.3
3 h	5.7	0.65	0.051	0.40	0.020	0.12	0.007	8.9
4 h	5.1	0.57	0.027	0.33	0.010	0.13	0.017	7.9
5 h	4.8	0.46	0.033	0.30	0.010	0.10	0.010	7.2
6 h	4.3	0.40	0.039	0.26	0.023	0.093	0.010	6.4
Experiment D (473 K)								
1 h	2.6	0.45	0.030	0.37	0.020	0.17	-*	5.4
2 h	2.4	0.37	0.027	0.28	0.023	0.11	-*	4.5
3 h	1.9	0.29	0.020	0.21	0.003	0.11	-*	3.6
4 h	1.8	0.27	0.017	0.19	0.020	0.074	-*	3.3

‡ 1 h TOFs discarded because of slow temperature adjustment

* TOF below quantification limit

References

[1] M. Rößler, P. Geng, J. Wintterlin, "A high-pressure scanning tunneling microscope for studying heterogeneous catalysis," *Rev. Sci. Instrum.* **2005**, *76*, 023705, https://doi.org/10.1063/1.1841951.

[2] K. M. Golder, B. Böller, G. Stienen, J. Sickerling, J. Wintterlin, "A highly sensitive gas chromatograph for in situ and operando experiments on catalytic reactions," *Rev. Sci. Instrum.* **2021**, *92*, 124103, https://doi.org/10.1063/5.0068021.

[3] G. Kresse, J. Furthmüller, "Efficient iterative schemes for ab initio total-energy calculations using a plane-wave basis set," *Phys. Rev. B* **1996**, *54*, 11169-11186, https://doi.org/10.1103/PhysRevB.54.11169.

[4] P. E. Blöchl, "Projector augmented-wave method," *Phys. Rev. B* **1994**, *50*, 17953-17979, https://doi.org/10.1103/PhysRevB.50.17953.

[5] B. Hammer, L. B. Hansen, J. K. Nørskov, "Improved adsorption energetics within density-functional theory using revised Perdew-Burke-Ernzerhof functionals," *Phys. Rev. B* **1999**, *59*, 7413-7421, https://doi.org/10.1103/PhysRevB.59.7413.

[6] S. Grimme, J. Antony, S. Ehrlich, H. Krieg, "A consistent and accurate ab initio parametrization of density functional dispersion correction (DFT-D) for the 94 elements H-Pu," *J. Chem. Phys.* **2010**, *132*, 154104, https://doi.org/10.1063/1.3382344.

[7] S. Grimme, "Density functional theory with London dispersion corrections," *Wiley Interdiscip. Rev.: Comput. Mol. Sci.* **2011**, *1*, 211-228, https://doi.org/10.1002/wcms.30.

[8] S. Grimme, A. Hansen, J. G. Brandenburg, C. Bannwarth, "Dispersion-Corrected Mean-Field Electronic Structure Methods," *Chem. Rev.* **2016**, *116*, 5105-5154, https://doi.org/10.1021/acs.chemrev.5b00533.

[9] D. Mahlberg, S. Sakong, K. Forster-Tonigold, A. Groß, "Improved DFT Adsorption Energies with Semiempirical Dispersion Corrections," *J. Chem. Theory Comput.* **2019**, *15*, 3250-3259, https://doi.org/10.1021/acs.jctc.9b00035.

[10] A. Banerjee, V. Navarro, J. W. M. Frenken, A. P. van Bavel, H. P. C. E. Kuipers, M. Saeys, "Shape and Size of Cobalt Nanoislands Formed Spontaneously on Cobalt Terraces during Fischer–Tropsch Synthesis," *J. Phys. Chem. Lett.* **2016**, *7*, 1996-2001, https://doi.org/10.1021/acs.jpclett.6b00555.

[11] M. W. Chase Jr., "NIST-JANAF Thermochemical Tables," *J. Phys. Chem. Ref. Data* **1998**, *Monograph 9*, 1-1951.

[12] B. Böller, K. M. Durner, J. Wintterlin, "The active sites of a working Fischer–Tropsch catalyst revealed by operando scanning tunnelling microscopy," *Nat. Catal.* **2019**, *2*, 1027-1034, https://doi.org/10.1038/s41929-019-0360-1.

[13] C. J. Weststrate, I. M. Ciobîcă, A. M. Saib, D. J. Moodley, J. W. Niemantsverdriet, "Fundamental issues on practical Fischer–Tropsch catalysts: How surface science can help," *Catal. Today* **2014**, *228*, 106-112, https://doi.org/10.1016/j.cattod.2013.11.042.
